# Nucleus accumbens medium spiny neurons subtypes signal both reward and aversion

**DOI:** 10.1038/s41380-019-0484-3

**Published:** 2019-08-28

**Authors:** Carina Soares-Cunha, Nivaldo A. P. de Vasconcelos, Bárbara Coimbra, Ana Verónica Domingues, Joana M. Silva, Eduardo Loureiro-Campos, Rita Gaspar, Ioannis Sotiropoulos, Nuno Sousa, Ana João Rodrigues

**Affiliations:** 1 Life and Health Sciences Research Institute (ICVS), School of Medicine, University of Minho, Braga, Portugal; 2 ICVS/3B’s-PT Government Associate Laboratory, Braga/Guimarães, Portugal; 3 Physics Department, Federal University of Pernambuco (UFPE), Recife, Pernambuco 50670-901 Brazil; 4 Department of Biomedical Engineering, Federal University of Pernambuco (UFPE), Recife, Pernambuco, 50670-901 Brazil; 5 Coimbra Institute for Clinical and Biomedical Research (iCBR), Faculty of Medicine, University of Coimbra, Coimbra, Portugal; 6 Center for Innovation in Biomedicine and Biotechnology (CIBB), University of Coimbra, Coimbra, Portugal; 7 Clinical Academic Center-Braga (2CA), Braga, Portugal

**Keywords:** Neuroscience, Addiction, Depression

## Abstract

Deficits in decoding rewarding (and aversive) signals are present in several neuropsychiatric conditions such as depression and addiction, emphasising the importance of studying the underlying neural circuits in detail. One of the key regions of the reward circuit is the nucleus accumbens (NAc). The classical view on the field postulates that NAc dopamine receptor D1-expressing medium spiny neurons (D1-MSNs) convey reward signals, while dopamine receptor D2-expressing MSNs (D2-MSNs) encode aversion. Here, we show that both MSN subpopulations can drive reward and aversion, depending on their neuronal stimulation pattern. Brief D1- or D2-MSN optogenetic stimulation elicited positive reinforcement and enhanced cocaine conditioning. Conversely, prolonged activation induced aversion, and in the case of D2-MSNs, decreased cocaine conditioning. Brief stimulation was associated with increased ventral tegmenta area (VTA) dopaminergic tone either directly (for D1-MSNs) or indirectly via ventral pallidum (VP) (for D1- and D2-MSNs). Importantly, prolonged stimulation of either MSN subpopulation induced remarkably distinct electrophysiological effects in these target regions. We further show that blocking κ-opioid receptors in the VTA (but not in VP) abolishes the behavioral effects induced by D1-MSN prolonged stimulation. In turn, blocking δ-opioid receptors in the VP (but not in VTA) blocks the behavioral effects elicited by D2-MSN prolonged stimulation. Our findings demonstrate that D1- and D2-MSNs can bidirectionally control reward and aversion, explaining the existence of controversial studies in the field, and highlights that the proposed striatal functional opposition needs to be reconsidered.

## Introduction

Daily, individuals assign emotional/motivational valence to otherwise neutral stimuli, by determining whether they are positive/rewarding and should be approached, or are negative/aversive and should be avoided. One crucial brain circuit in this process is the mesolimbic reward pathway, which comprises dopaminergic projections from the ventral tegmental area (VTA) to the nucleus accumbens (NAc). This circuit has been implicated in the recognition of rewards in the environment, which elicit approach and consummatory behavior and attribute motivational value to objects that signal its delivery/presence [1–4]. Besides this traditional role of VTA–NAc projections in rewarded behaviors, these also mediate/signal aversive stimuli, which is crucial to avoid threats and ensure survival [5–7]. Importantly, dysfunction of the reward circuit has been implicated in several neuropsychiatric disorders, being addiction one of the most studied [8].

Several studies suggest that the VTA is composed of anatomically and functionally heterogeneous dopamine neuronal subpopulations with different axonal projections, which may explain its role in both reward and aversion [5, 6, 9]. Similarly, VTA neurons also receive different inputs; for example, activation of laterodorsal tegmentum inputs to the VTA elicit reward, whereas from the lateral habenula induce aversion [6].

One of the core regions decoding rewarding/aversive signals from the VTA is the NAc [10]. These signals (mostly dopaminergic) act through two distinct neuronal populations of GABAergic medium spiny neurons (MSNs), segregated into those expressing dopamine receptor D1 (D1-MSNs) or dopamine receptor D2 (D2-MSNs). D1-MSNs project directly to output nuclei of the basal ganglia, namely the VTA (direct pathway), but can also project indirectly through the ventral pallidum (VP) (indirect pathway). D2-MSNs project exclusively indirectly to output nuclei of the basal ganglia through the VP [11, 12].

The canonical view on striatal function is that D1-MSNs encode positive valence/reward, whereas D2-MSNs encode negative/aversive responses [13–16]. In the dorsomedial striatum, D1-MSN optogenetic stimulation induces persistent reinforcement, while D2-MSN stimulation induces transient punishment [16]. In the same direction, stimulation of NAc D1-MSNs enhances cocaine-mediated conditioning, whereas optical stimulation of D2-MSNs suppresses it [15]. Yet, recent studies suggest that these two subpopulations may exert a concurrent action in reward-related behaviors [17–21]. For example, in dorsolateral striatum, both MSN subpopulations are involved in positive reinforcement, but support different action strategies [21]. We have shown that activation of either type of NAc MSNs during cue exposure strongly enhances motivational drive toward natural rewards [18, 20], suggesting that D2-MSNs do not exclusively modulate negative stimuli.

Our hypothesis to explain this conundrum was that both D1- and D2-MSNs can convey positive and negative stimuli through different patterns of activation and consequent changes in downstream target regions, such as the VP, to which both subpopulations project, or the VTA, only innervated by D1-MSNs. Our results show that both MSN subpopulations can drive reward and aversion and differentially modulate cocaine conditioning. These divergent behavioural outputs are associated with MSN pattern of stimulation and consequent downstream electrophysiological effects.

## Materials and methods

Methods are described in more detail in Supplementary information.

### Animals

Male and female D1-cre (line EY262, Gensat.org) and D2-cre (line ER44, Gensat.org) mice were used. All animals were maintained under standard laboratory conditions: artificial 12 h light/dark cycle, temperature of 21 ± 1 °C and 60% relative humidity; mice were given standard diet and water ad libitum. All behavioral experiments were performed during the light period of the cycle.

Health monitoring was performed according to FELASA guidelines. All procedures were conducted in accordance with European Union regulations (Directive 2010/63/EU). Animal facilities and the people directly involved in animal experiments were certified by the Portuguese regulatory entity DGAV. All protocols were approved by the Ethics Committee of the Life and Health Sciences Research Institute and by DGAV (#19074).

### Constructs, virus injection, and cannula implantation for optogenetic manipulation

Cre-inducible AAV5/EF1a-DIO-hChR2(H134R)-eYFP, AAV5/EF1a-DIO-eNpHR-eYFP, and AAV5/EF1a-DIO-eYFP were obtained directly from the UNC Gene Therapy Center (University of North Carolina, NC, USA). AAV5 vectors titers were 3.7–6 × 10^12^ viral molecules/ml as determined by dot blot.

Stereotaxic surgery was performed as described in Supplementary information. For optical stimulation in the NAc, 500 nl of virus was unilaterally injected into the NAc of D1- and D2-cre mice (coordinates from bregma [22]: +1.3 mm anteroposterior (AP), +0.9 mm mediolateral (ML), −4.0 mm dorsoventral (DV)), and an optic fiber was implanted using the same coordinates (with the exception of DV: −3.9 mm). For optical stimulation + drug delivery in terminals, the guide cannula was implanted in the VTA (−3.2 mm AP, +0.5 mm ML, and 4.5 mm DV) or the VP (0.1 mm AP, +1.6 mm ML, and −3.9 mm DV).

Optical manipulation was performed using a 473 nm (ChR2) or 589 nm (NpHR) DPSS lasers, which were controlled using a pulse generator (Master-8; AMPI, New Ulm, MN, USA). Stimulation parameters: brief: 1 s, 12.5 ms pulses at 40 Hz; prolonged: 1 s, 12.5 ms pulses at 40 Hz; inhibition: 10 s constant light.

### Place preference tests

The conditioned place preference (CPP) and Real-time place preference (RTPP) protocols were previously described [15, 23, 24]; and are described in detail in Supplementary material. Briefly, in the CPP with optical stimulation, animals were exposed to one pretest session (15 min), two conditioning sessions (30 min; 1 day with and 1 day without stimulation), and one posttest session (15 min). In the CPP with cocaine (5 mg/kg) + optical stimulation, animals were exposed to one pretest session (20 min), 2 days of conditioning—morning session with cocaine + optical stimulation and afternoon session with saline + no optical stimulation (30 min each session), and one posttest session (20 min).

In the RTPP, a single 15 min session was performed, in which one of the chambers was associated with optical stimulation.

### In vivo single cell electrophysiology

In brief, a recording electrode coupled with a fiber optic patch cable was placed in the NAc (+1.3 mm AP, +0.9 mm ML, and 3.5 to 4.2 mm DV), VP (−0.12 mm AP, +1.6 mm ML, and −3.5 to 4 mm DV) and in the VTA (−3.2 mm AP, +0.5 mm ML, and 4 to 4.8 mm DV). Spikes of single neurons were recorded and further analyzed. All details in Supplementary information.

### Drugs

Drugs or vehicle were delivered 20 min before animals performed the CPP test, through a fluid system chronically implanted in the VP or VTA. Naltrindole 0.1 μg (Nal, δ-opioid receptor (DOR) antagonist, Sigma) and norbinaltorphimine 1 μg (nor-BNI, κ-opioid receptor (KOR) antagonist, Sigma) were administered.

### Immunofluorescence

Paraformaldehyde-fixed sections were incubated with specific antibodies against GFP (1:500, Ab6673, Abcam), D1R (1:200, NB110-60017, Novus), or D2R (1:400, sc-5303, Santa Cruz Biotechnology). Positive cells within the brain regions of interest were quantified by confocal microscopy. Details in supplementry material.

### Statistical analysis

Prior to any statistical comparison between groups, normality tests (Shapiro–Wilks (S–W)) were performed for all data analysed. Results are presented as mean ± SEM. Statistical details can be found throughout the results description; these include the value of the statistical tests used and exact *p*-value. Statistical results for optogenetic prolonged stimulation using a 30 min protocol are presented in Supplementary Tables 1 and 2. The *n* for each experiment is indicated in figures’ legends. More details in Supplementary information.

## Results

### Brief optical stimulation of NAc D1- or D2-MSNs induces preference

**Fig. 1:**
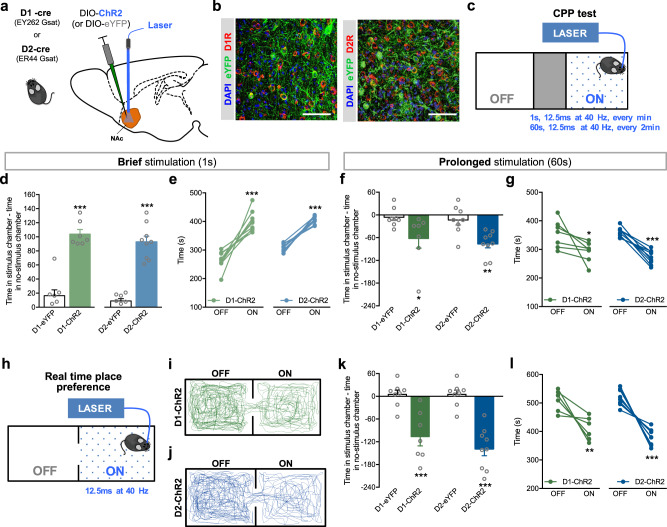
Bidirectional effect of NAc D1- and D2-MSNs in reinforcement. **a** Strategy used for optogenetic manipulation. A cre-dependent ChR2 or eYFP was injected unilaterally in the NAc of D1-cre or D2-cre transgenic mice. **b** Representative immunofluorescence of eYFP and D1R (left panel) or D2R (right panel). **c** Schematic representation of optogenetic stimulation parameters in the CPP test (1 s or 60 s of 12.5 ms pulses at 40 Hz). **d** Brief (1 s) optical stimulation of NAc D1- or D2-MSNs induces place preference (*n*_D1-eYFP_ = 8, *n*_D1-ChR2_ = 7; *n*_D2-eYFP_ = 8, *n*_D2-ChR2_ = 9). **e** Increased preference for the stimulus-associated chamber (ON) in all D1-ChR2 and D2-ChR2 animals. **f** Prolonged (60 s) optical stimulation of NAc D1- or D2-MSNs induces aversion (*n*_D1-eYFP_ = 8, *n*_D1-ChR2_ = 7; *n*_D2-eYFP_ = 8, *n*_D2-ChR2_ = 9). **g** Aversion to the ON chamber in D1-ChR2 and D2-ChR2 animals. **h** Schematic representation of the real-time preference apparatus. Whenever animals are on the “ON chamber”, they receive optogenetic stimulation (12.5 ms pulses at 40 Hz) that ceases only when animals cross to the OFF side. Representative track of (**i**) D1-ChR2 and D1-eYFP animals, and (**j**) D2-ChR2 and D2-eYFP animals. **k**, **l** Both D1-ChR2 and D2-ChR2 mice spent less time in the stimulus-associated box (*n*_D1-eYFP_ = 7, *n*_D1-ChR2_ = 8; *n*_D2-eYFP_ = 8, *n*_D2-ChR2_ = 9). *p < 0.05, **p < 0.01, ***p < 0.001. Data are represented as mean ± SEM

To date, there is still controversy regarding which NAc MSN subpopulation encodes reward and aversion, so we used optogenetics to specifically manipulate NAc D1- or D2-MSNs activity. D1- or D2-cre mice were injected in the NAc with AAV5 containing a cre-dependent channelrhodopsin (ChR2, optical stimulation), halorhodopsin (NpHR, optical inhibition), or eYFP (control group) (Fig. 1a). This approach successfully transfected ~60% of NAc D1^+^ cells (eYFP^+^/D1R^+^ cells) and ~50% of D2^+^ cells (eYFP^+^/D2R^+^ cells) (Fig. 1b; Supplementary Fig. 1a).

We next evaluated the reinforcing properties of NAc D1- or D2-MSN modulation, using an unbiased CPP paradigm, a noncontingent conditioning paradigm (experimenter induced), in which one of the chambers was associated with optical stimulation (or inhibition) (Fig. 1c).

D1-MSN brief optical stimulation (1 s stimulus—40 pulses of 12.5 ms at 40 Hz, every minute) induced preference for the stimulus-associated (ON) chamber (Fig. 1d, *U* = 0.0, *p* = 0.0003; Fig. 1e, *t*_6_ = 6.2, *p* = 0.0008), in agreement with the proposed proreward role of this subpopulation [15, 16]. Surprisingly, brief optogenetic stimulation of D2-MSNs also induced preference for the stimulus-associated chamber (Fig. 1d; *t*_15_ = 9.0, *p* < 0.000), with all animals showing preference for the ON side (Fig. 1e; *t*_8_ = 12.4, *p* < 0.000). D1-eYFP and D2-eYFP control mice showed no preference (Supplementary Fig. 1b).

Optogenetic inhibition of D1-MSNs (10 s of constant light at 5 mW, every minute) triggered aversion to the ON chamber (Supplementary Fig. 2; *t*_8_ = 3.6, *p* = 0.0065). A similar result was observed with D2-MSN optical inhibition, since D2-eNpHR mice showed aversion to the stimulus chamber (*t*_8_ = 3.3, *p* = 0.0296).

### Prolonged optical stimulation of NAc D1- or D2-MSNs induces aversion

Given that our results differ from the study by Lobo et al., which showed that D1- or D2-MSN optogenetic activation (3 min at 10 Hz) did not induce place preference (a finding that we replicated–Supplementary Fig. 3); and that D2-MSN activation even abolished cocaine conditioning effects [15], we hypothesized that distinct patterns of stimulation led to different behavioral outcomes, explaining the discrepancies between studies.

Hence, we performed the CPP with prolonged stimulation of NAc MSNs—60 s, 12.5 ms pulses at 40 Hz, stimulus given every other minute. Prolonged stimulation of D1-MSNs induced aversion, since D1-ChR2 mice decreased their preference for the ON chamber (Fig. 1f, *U* = 8.0, *p* = 0.0205; Fig. 1g, *t*_6_ = 2.5, *p* = 0.0485). Prolonged stimulation of D2-MSNs also induced aversion to the stimulus-associated chamber (Fig. 1f, *t*_15_ = 3.6, *p* = 0.0024; Fig. 1g, *t*_8_ = 8.3, *p* < 0.000).

We also tested other optical stimulation protocols; 10 Hz stimulation of either subpopulation did not induce preference, as expected [15, 25]; whereas 20 Hz stimulation induced a similar result as 40 Hz stimulation (Supplementary Fig. 4).

Because our results were surprising, we added a second behavioral readout to evaluate the impact of D1- and D2-MSN stimulation in reward/aversion. So, we used the RTPP paradigm (Fig. 1h), which also measures the reinforcing properties of a stimulus, but it is dependent on subject’s choice (contingent). Optical stimulation (12.5 ms light pulses at 40 Hz) occurred whenever the animal was in the ON box.

D1-MSN stimulation induced aversion to the ON side (Fig. 1i, k, l; *t*_6_ = 4.3, *p* = 0.0051). Similarly, D2-MSN optical stimulation also induced aversion to the ON side (Fig. 1j–l; *t*_8_ = 7.5, *p* < 0.001). As all animals were subjected on average to 60 s of stimulation per entry in the ON chamber (Supplementary Fig. 1c, d), these results corroborate the prolonged stimulation data of the CPP test.

Neither brief nor prolonged optical stimulation induced significant differences in locomotion (Supplementary Fig. 5).

### Differential effect of D1- and D2-MSN optical stimulation on cocaine CPP

**Fig. 2:**
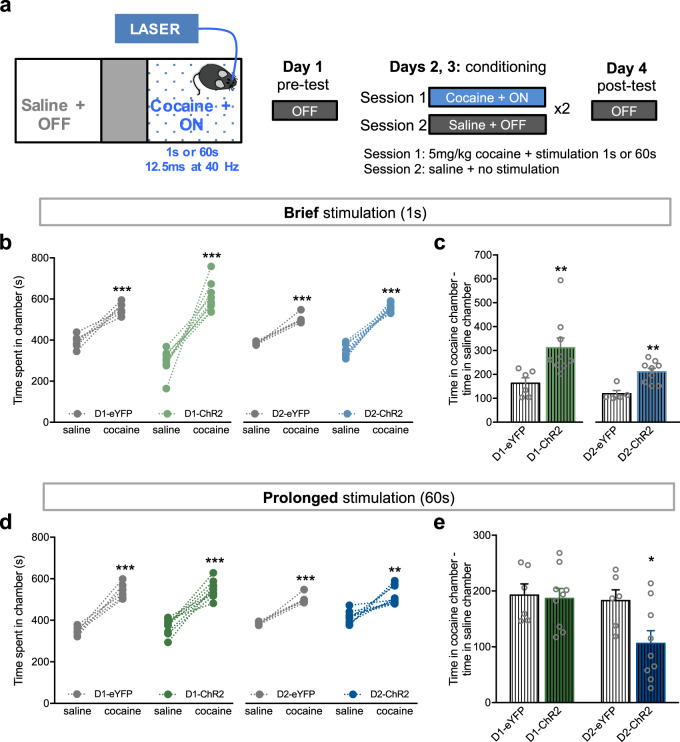
Brief and prolonged D1- and D2-MSN stimulation differentially modulate cocaine conditioning. **a** Representation of the CPP cocaine 5 mg/kg + optical stimulation (1 s or 60 s) paradigm. **b** Cocaine induces preference in all groups as expected; and brief optical activation of D1- or D2-MSNs further enhances cocaine reinforcing effects (**c**). In comparison with eYFP groups, D1-ChR2 or D2-ChR2 spend more time in the cocaine + brief stimulus chamber (*n*_D1-eYFP_ = 6, *n*_D1-ChR2_ = 9; *n*_D2-eYFP_ = 5, *n*_D2-ChR2_ = 9). **d** Cocaine induces preference in all groups. Prolonged optical activation of D1-MSNs does not change the reinforcing properties of cocaine, whereas D2-MSN prolonged stimulation decreases cocaine reward (**e**) (*n*_D1-eYFP_ = 6, *n*_D1-ChR2_ = 9; *n*_D2-eYFP_ = 6, *n*_D2-ChR2_ = 9). **p < 0.01, ***p < 0.001. Data are represented as mean ± SEM

Our data contrasted with the results of optical activation of D1- or D2-MSN in the context of cocaine conditioning [15]. Thus, we performed the classic CPP using cocaine (5 mg/kg) as the conditioned stimulus together with brief or prolonged stimulation of NAc D1- or D2-MSNs (Fig. 2a).

As anticipated, ChR2 and eYFP groups were conditioned by cocaine (Fig. 2b; saline vs. cocaine: D1-eYFP, *t*_5_ = 7.2, *p* = 0.0008; D1-ChR2, *t*_8_ = 7.7, *p* < 0.0001; D2-eYFP, *t*_4_ = 9.0, *p* = 0.0008; D2-ChR2, *t*_8_ = 13.9, *p* < 0.0001). Interestingly, brief optical stimulation of D1- or D2-MSNs significantly heightened cocaine preference as observed in the difference of time spent in cocaine chamber—saline chamber (Fig. 2c; D1-eYFP vs. D1-ChR2, *U* = 2.0, *p* = 0.0016; D2-eYFP vs. D2-ChR2, *U* = 3.0, *p* = 0.007). Conversely, prolonged D1-MSN stimulation had no effect in enhancing cocaine conditioning (Fig. 2e; *t*_13_ = 0.2, *p* = 0.8278). Prolonged D2-MSN stimulation decreased cocaine-conditioning effects (Fig. 2e; *t*_13_ = 2.4, *p* = 0.0332), as previously reported with 3 min 10 Hz stimulation of this subpopulation [15].

Cocaine induced the same locomotion effects in all groups (Supplementary Fig. 6).

### NAc electrophysiological correlates

**Fig. 3:**
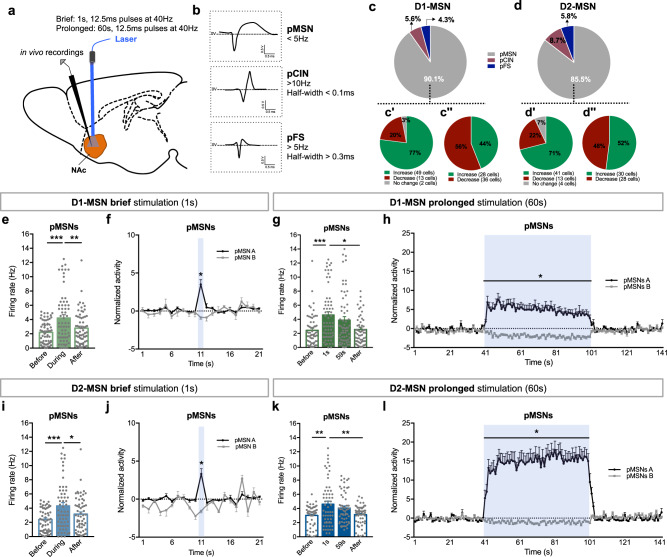
Electrophysiological response of NAc neurons to brief or prolonged stimulation. **a** Schematic representation of NAc electrophysiological recordings. **b** NAc neurons were separated into three categories according to firing rate and waveform characteristics: putative medium spiny neurons (pMSNs), cholinergic interneurons (pCINs), and fast spiking GABAergic interneurons (pFS). **c** In D1-cre stimulated mice, 90.1% of recorded cells were classified as pMSNs (64/71 cells), 5.6% as pCINs, and 4.3% as pFS interneurons. During D1-MSNs brief (**c′**) or prolonged (**c″**) stimulation, 77% and 44% of the pMSNs increased their activity, respectively. **d** In D2-cre stimulated mice, 85.5% of recorded cells were pMSNs (59/69 cells), 8.7% pCINs, and 5.8% pFS interneurons. D2-MSNs brief (**d′**) or prolonged (**d″**) stimulation increased the activity in 71% and 52% of the pMSNs, respectively. **e** D1-MSNs brief stimulation increased average firing rate of MSNs. **f** Temporal variation of the normalized activity of pMSNs that increase (pMSN A) and decrease (pMSN B) activity during the stimulation period (blue). **g** D1-MSNs prolonged stimulation increased average firing rate of MSNs. **h** Temporal variation of the normalized activity of pMSNs showing the distinct pattern of response during stimulation (blue). **i** D2-MSNs brief stimulation increased the average firing rate of MSNs. **j** Temporal variation of the normalized activity of pMSNs showing the response during stimulation. **k** Prolonged D2-MSNs stimulation increased average firing rate of MSNs throughout the stimulation period. **l** Temporal variation of the normalized activity of pMSNs showing the response during stimulation. *p < 0.05. Data are represented as mean ± SEM

Our behavioral data indicated that D1- and D2-MSNs drive both reward and aversion, depending on their stimulation period. To understand the functional impact of different periods of stimulation, we performed in vivo single unit electrophysiological recordings in the NAc (Fig. 3a). Different NAc neuronal populations—putative MSNs (pMSN), cholinergic interneurons (pCIN), and fast-spiking GABAergic interneurons (pFS), were identified based on characteristic waveforms and basal firing rate [26, 27] (Fig. 3a, b).

Brief stimulation of both D1- and D2-MSNs increased activity of the majority of MSNs (77% and 71%, respectively). Prolonged stimulation elicited a distinct response, with more MSNs decreasing activity during stimulation (56% for D1- and 48% for D2-MSN prolonged stimulation) (Fig. 3c, d).

D1-MSN brief optical stimulation increased average firing rate of pMSNs (Fig. 3e; *F*_2,126_ = 18.6, *p* < 0.0001, post hoc before vs. during *p* < 0.0001); firing rate returned to basal levels after stimulation period (the same was observed with a 30 min protocol; Supplementary Fig. 7). This effect was also shown by the temporal variation of the activity of the cells (Fig. 3f). MSNs were divided into those that increase activity (>20% from of baseline) during stimulation—pMSNs A, and other type of response—pMSNs B (Fig. 3f).

Despite the fact that there is a higher percentage of MSNs decreasing firing rate in the prolonged D1-MSN stimulation in comparison with brief stimulation, we observe the same net effect, i.e., there is a net increase in average firing rate of MSNs (Fig. 3g; *F*_3,189_ = 10.6, *p* < 0.0001, post hoc before vs. during (1 s) *p* < 0.000, post hoc before vs. during (59 s) *p* = 0.007). These neurons return to baseline activity after stimulation period (Fig. 3h; pMSNs A: KS = 1.0, *p* < 0.001; pMSN B: KS = 0.9, *p* < 0.001; Supplementary Fig. 8).

Regarding D2-MSN optical stimulation, a significant increase in MSN firing rate was also observed with 1 s stimulation (Fig. 3i; *F*_2,116_ = 13.9, *p* < 0.000, post hoc before vs. during *p* < 0.000; Fig. 3j; pMNS A, *p* < 0.000; pMSN B, *p* = 0.05; Supplementary Fig. 7). Prolonged stimulation of D2-MSNs resulted in increased average firing rate (Fig. 3k; *F*_3,174_ = 8.8, *p* = 0.001, post hoc before vs. during (1 s) *p* = 0.0056, post hoc before vs. during (59 s) *p* = 0.0013), also evident in the temporal variation of the activity of these neurons (Fig. 3l; pMSN A, KS = 1.0, *p* < 0.001; pMSN B, KS = 0.9, *p* < 0.001; Supplementary Fig. 8).

No major differences in the average firing rate of pCINs and pFSs were found, though these results need to be interpreted carefully because of the low number of neurons recorded (Supplementary Fig. 9).

### Electrophysiological effects in the VP and VTA in response to D1-MSN stimulation

**Fig. 4:**
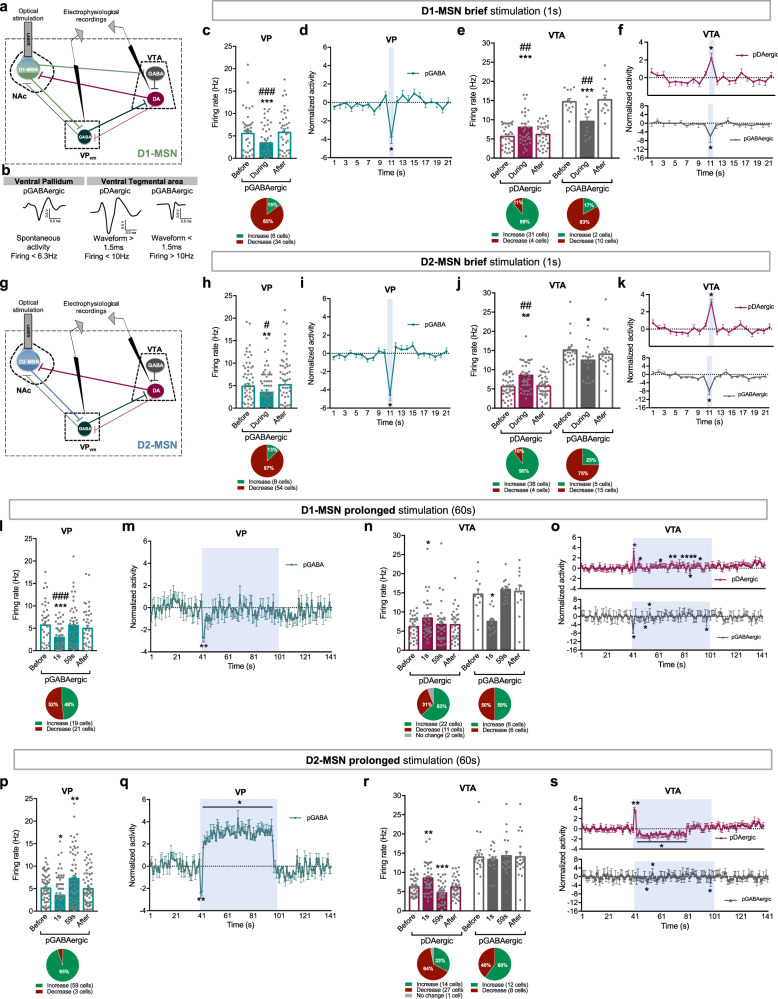
Distinct electrophysiological responses of VP and VTA during brief or prolonged MSNs stimulation. **a** NAc D1-MSN circuitry. **b** Representative waveform of a VP pGABAergic neuron, and VTA pGABAergic and pDAergic neurons. Pie charts represent the percentage of each type of response to stimulus; bar graphs represent net firing rate before, during and after optogenetic stimulation; blue stripe in scatterplots represents optogenetic stimulus. **c** Brief D1-MSNs optical stimulation decreases the net firing rate of VP pGABAergic neurons. **d** Temporal variation of VP activity; note the decrease in activity during optical stimulation (blue). **e** The same stimulation increases the net activity of VTA pDAergic neurons; conversely, pGABAergic neurons decrease activity. **f** Temporal variation of VTA neurons activity. **g** NAc D2-MSNs circuitry. Brief D2-MSNs optical stimulation induces a similar response in VP and VTA regions as D1-MSNs brief stimulation (**h**–**k**). **l** Prolonged D1-MSNs optical stimulation decreases the average VP firing rate in the first second of stimulation; then net activity normalizes to baseline during the rest of the stimulation. **m** Temporal variation of VP activity. **n** Prolonged D1-MSNs stimulation induced an increase in the average firing rate of VTA pDAergic neurons in the first second of stimulation, contrary to pGABAergic neurons, that decreased activity. **o** Temporal variation of the activity of VTA neurons. Note the opposing patterns of activity in pDAergic and pGABAergic neurons. **p** Prolonged optical stimulation of D2-MSNs decreases the activity of VP neurons in the first second of stimulation, and increases it during the rest of the stimulation. **q** Temporal variation of VP activity; note the decrease in activity during the first seconds of optical stimulation, and the increase thereafter. **r** This same stimulation increased the average firing rate of VTA pDAergic neurons in the first second of stimulation, and decreased their activity after. No changes were found in the activity of VTA pGABAergic neurons. **s** Temporal variation of the activity of VTA neurons. “*” Before vs. during; “#” during vs. after; *p < 0.05, ** or ^##^*p* < 0.01, ***p < 0.001. Data are represented as mean ± SEM

Our results suggested that brief or prolonged optogenetic stimulation of either subpopulation did not elicit a remarkably distinct electrophysiological response in the NAc, so downstream regions could be mediating the divergent behavioral effects. Thus, we evaluated the neuronal activity of the VTA, directly innervated by D1-MSNs, and the VP [12, 28, 29] innervated by both MSN subpopulations [12]. VP GABAergic neurons also provide a tonic inhibitory input to the VTA [12, 30–32] (Fig. 4a).

Brief stimulation of D1-MSNs significantly decreased the average firing rate of 85% of identified VP putative GABAergic (pGABAergic) neurons (Fig. 4c, d; *F*_2,78_ = 17.4, *p* < 0.001, post hoc before vs. during *p* = 0.0003; Supplementary Figs. 7, 10). Accordingly, temporal variation of VP activity shows a decrease in activity during optical stimulation (Fig. 4d; baseline vs. stimulus, *p* < 0.000).

In the VTA, cells were separated in putative dopaminergic (pDAergic) and pGABAergic neurons (Fig. 4b), according to their firing rate and waveform duration [33–35]. Brief stimulation of D1-MSNs increased average firing rate of 89% of pDAergic neurons; in contrast, 83% of pGABAergic neurons decreased activity (Fig. 4e, f; pDAergic: *F*_2,66_ = 8.8, *p* = 0.0023, post hoc before vs. during *p* = 0.0025; pGABAergic: *F*_2,22_ = 12.9, *p* = 0.0019, post hoc before vs. during *p* = 0.0098; Supplementary Figs. 7, 10). Temporal variation revealed an opposite response of VTA pDAergic and pGABAergic neurons (Fig. 4f; pDAergic and pGABAergic: baseline vs. stimulus, *p* < 0.000; Supplementary Fig. 7).

Conversely, prolonged D1-MSN stimulation decreased firing rate of VP neurons during the first 2 s of stimulation (Fig. 4l, m; *F*_3,159_ = 13.9, *p* < 0.000, post hoc before vs. during (1 s) *p* = 0.0002); those neurons return to baseline activity after that (Fig. 4m; KS = 0.6, *p* < 0.000; Supplementary Fig. 8).

In the VTA, prolonged D1-MSN activation led to an increase in average firing rate of pDAergic neurons during the first second of stimulation (Fig. 4n, o; *F*_3,99_ = 3.9, *p* = 0.0203, post hoc before vs. during (1 s) *p* = 0.0233; Supplementary Fig. 8), and no major changes thereafter. When analyzing the response throughout time, pDAergic neurons present a marked increase in activity in the first second of stimulation that then decrease to levels comparable with baseline (with the exception of few time points) as stimulation continues (Fig. 4o; KS = 0.6, *p* < 0.000). Conversely, VTA pGABAergic neurons activity was decreased in the first second of stimulation (Fig. 4n, o; *F*_3,33_ = 10.5, *p* = 0.0037, post hoc before vs. during (1 s) *p* = 0.0055), and then normalized, except for some time points (Fig. 4o; KS = 0.4, *p* = 0.002).

Altogether, this data indicates that brief D1-MSN stimulation decreases VP activity and enhances VTA dopaminergic activity. Prolonged stimulation induces similar effects but only during the first seconds of stimulation, and then the activity of these brain regions *quasi* normalizes.

### Electrophysiological effects in the VP and VTA in response to D2-MSN stimulation

NAc D2-MSNs do not project to the VTA directly, but are able to control midbrain activity indirectly through the VP [12] (Fig. 4g).

Accordingly, brief stimulation of D2-MSNs decreased activity of 87% of VP neurons (Fig. 4h, i; *F*_2,122_ = 9.7, *p* = 0.0014, post hoc before vs. during *p* = 0.0109). Temporal variation of VP neurons shows this effect time-locked to optical stimulation (Fig. 4i; baseline vs. stimulus, *p* < 0.000; Supplementary Fig. 7). This occurred prior to the increase in activity of 90% of VTA pDAergic neurons (Fig. 4j, k: *F*_2,80_ = 11.9, *p* = 0.0010, post hoc before vs. during *p* = 0.0025; Fig. 4k: baseline vs. stimulus, *p* < 0.000). Latency to fire of pDAergic neurons was 139.5 ms, indicative of polysynaptic modulation (Supplementary Fig. 10). The majority of pGABergic neurons presented a decrease in activity during stimulation, that was significantly different from baseline (Fig. 4j, k; *F*_2,57_ = 7.5, *p* = 0.0228, post hoc before vs. during *p* = 0.0273).

Contrariwise, prolonged optogenetic stimulation of D2-MSNs decreased average firing rate of VP neurons during the first 2 s of stimulation, and increased VP activity after that period (Fig. 4p, q; *F*_3,183_ = 16.9, *p* < 0.000, post hoc before vs. during (1 s) *p* = 0.0163, post hoc before vs. during (59 s) *p* = 0.0081; Supplementary Fig. 8). When analyzing the response throughout time, this increase in activity after the initial 2 s inhibition was marked (Fig. 4q; KS = 0.4, *p* < 0.000).

Regarding VTA, prolonged activation of D2-MSNs caused an increase in VTA pDAergic firing rate in the first second of stimulation and a decrease thereafter (Fig. 4r; *F*_3,120_ = 19.5, *p* < 0.000, post hoc before vs. during (1 s) *p* = 0.0037, post hoc before vs. during (59 s) *p* = 0.0003; Supplementary Fig. 8). This effect was particularly evident in the temporal analysis of VTA activity (Fig. 4s; KS = 0.4, *p* = 0.003). In contrast, prolonged D2-MSN stimulation did not cause major differences in pGABAergic net neuronal activity (Fig. 4r, s).

Our results indicate that D2-MSN brief stimulation leads to increase in VTA dopaminergic activity, caused by indirect inhibition of VP GABAergic neurons. Inversely, prolonged stimulation of D2-MSNs causes a significant increase in VP activity, which likely contributes for the decrease in VTA dopaminergic signaling (after the first seconds of stimulation).

### D1- and D2-MSN-induced place aversion is mediated by activation of distinct opioid receptors

We next explored the mechanism underlying the aversive effect induced by prolonged stimulation of MSNs. D1-MSNs express and corelease dynorphin (KOR ligand), whereas D2-MSNs express and corelease enkephalin (DOR ligand) [12, 36–38]. These opioids can have complex pre- and postsynaptic effects, and have been associated with reward and aversion [39, 40].

**Fig. 5:**
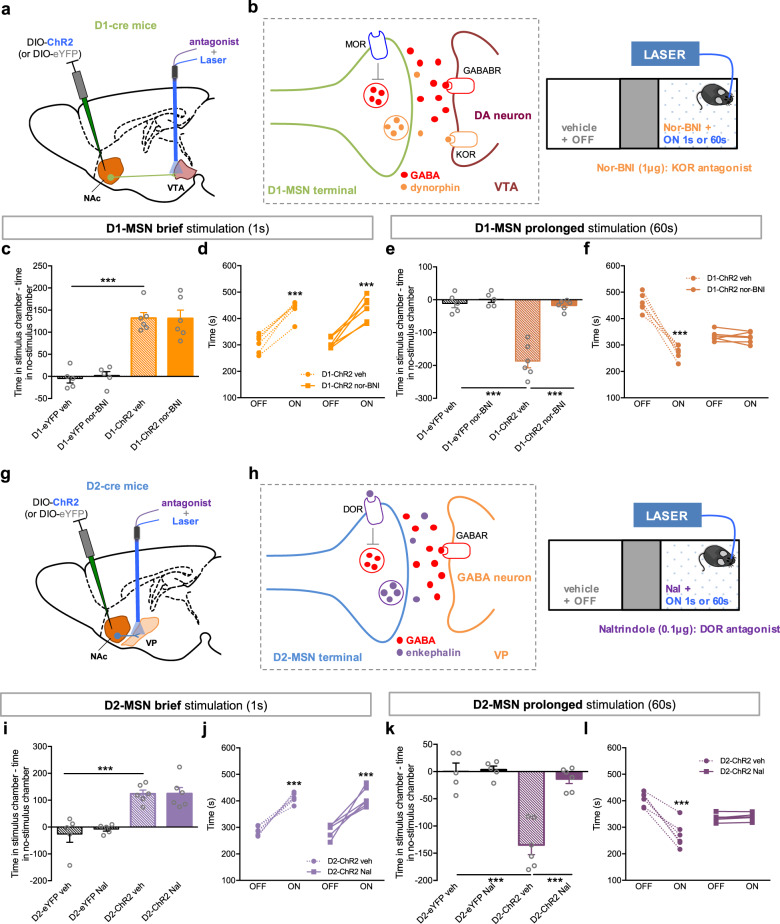
Aversion induced by prolonged MSN stimulation is mediated by opioids. **a** A Cre-dependent ChR2 was injected in the NAc of D1-cre mice and a guide cannula was placed in the VTA to allow injection of drugs and optical stimulation of D1-MSN terminals. **b** D1-cre mice were injected with nor-BNI (KOR antagonist, 1 μg/0.5 μl) in the VTA 20 min before the CPP conditioning session with brief or prolonged optical stimulation. In the VTA, KOR is mainly expressed in dopaminergic neurons. **c**, **d** Nor-BNI administration had no effect on D1-MSN brief stimulation-induced CPP (*n*_D1-eYFPveh_ = 5, *n*_D1-eYFPnor-BNI_ = 5, *n*_D1-ChR2veh_ = 6, *n*_D1-ChR2veh_ = 6). **e**, **f** Nor-BNI occluded D1-MSN prolonged optical stimulation-induced aversion (*n*_D1-eYFPveh_ = 5, *n*_D1-eYFPnor-BNI_ = 5, *n*_D1-ChR2veh_ = 6, *n*_D1-ChR2nor-BNI_ = 6). D1-ChR2 nor-BNI mice did not show preference for any chamber. **g** A Cre-dependent ChR2 was injected in the NAc of D2-cre mice and a guide cannula was placed in the VP to allow injection of drugs and optical stimulation of D2-MSN terminals. **h** D2-cre mice were injected with Nal (DOR- antagonist 0.1 μg/0.5 μl) in the VP 20 min before the CPP conditioning session with brief or prolonged optical stimulation. In the VP, DOR is expressed in D2-MSN terminals arising from the NAc. **i**, **j** Nal administration had no effect on D2-MSN brief stimulation-induced CPP (*n*_D2-eYFPveh_ = 5, *n*_D2-eYFPNal_ = 5, *n*_D2-ChR2veh_ = 6, *n*_D1-ChR2Nal_ = 6). **k**, **l** Nal occluded the effect of D2-MSN prolonged optical stimulation, blocking the aversive effect (*n*_D2-eYFPveh_ = 5, *n*_D2-eYFPNal_ = 5, *n*_D2-ChR2veh_ = 6, *n*_D2-ChR2Nal_ = 6). ***p < 0.001. Data are represented as mean ± SEM

We injected a cre-inducible ChR2 virus in the NAc of D1- or D2-cre mice, and implanted in the VTA or VP, respectively, a hybrid cannula that allows drug delivery and optical stimulation (Fig. 5a, g). We next performed the CPP test with optical stimulation of either MSN subpopulation in animals previously injected in the VTA or VP with KOR or DOR antagonists (Fig. 5b, h).

D1-MSN-VTA or D2-MSN-VP terminal stimulation recapitulated the effects of D1- or D2-MSNs soma stimulation, respectively (Fig. 5), supporting the involvement of VTA and VP downstream targets in the observed behavioral phenotype.

VTA injection of nor-BNI (KOR antagonist) had no impact in animals submitted to D1-MSN brief stimulation (Fig. 5c, d), but occluded the behavioral effects of prolonged stimulation since D1-ChR2 stimulated animals no longer manifest aversion to the ON side (Fig. 5e, f; D1-ChR2 veh vs. D1-ChR2 nor-BNI, *t*_10_ = 7.9, *p* = 0.000). As a control experiment, we also injected nor-BNI in the VP of D2-MSN prolonged stimulation group, and observed no effect (Supplementary Fig. 11).

Injection of naltrindole (DOR antagonist) in the VP did not affect D2-MSN brief stimulation-induced effects in behavior (Fig. 5i, j). Conversely, it abolished the aversive effect caused by prolonged D2-MSN optical stimulation (D2-ChR2 veh vs. D2-ChR2 Nal, *t*_10_ = 6.2, *p* = 0.0001). As anticipated, naltrindole injection in the VTA had no effect whatsoever in prolonged D1-MSN stimulation (Supplementary Fig. 11).

## Discussion

Here we show that optogenetic activation of NAc D1- or D2-MSNs (soma or terminals) induces reward or aversion, depending on the characteristics of MSN stimulation protocol. Brief stimulation induced positive reinforcement and enhanced cocaine conditioning; whilst prolonged stimulation of either subpopulation induced aversion. Importantly, we show that these distinct stimulation protocols elicit divergent electrophysiological responses in downstream targets, the VTA and VP.

The VTA-NAc pathway is crucial to integrate neural information from the cortex/thalamus and facilitate selection of actions that achieve reward and avoid aversive outcomes [11, 41, 42], thus it is not surprising that dysfunction of this pathway underlies some neuropsychiatric disorders. For example, depression and addiction are characterized by a marked dysfunction of NAc both in animal models and humans [43–48], which highlights the importance of studying this region in more detail.

Classical views on striatal function propose a dichotomous role for D1- and D2-MSNs (both in dorsal striatum and NAc) in encoding rewarding and aversive signals [49, 50]. In the dorsal striatum, optogenetic activation of D1-MSNs induces preference, whereas D2-MSN stimulation aversion [16]. Similarly, in the NAc, optogenetic activation of D1-MSNs enhanced cocaine conditioning, whereas D2-MSNs activation abolished cocaine effects [15], which lead to the assumption that these neurons also convey opposing valence information. Yet, recent evidence suggested that this view is too simplistic. For example, in dorsolateral striatum, both MSN subpopulations are involved in positive reinforcement, but support different action strategies [21]. In line with this, we have shown that brief optogenetic activation of D1- or D2-MSNs enhanced motivational drive toward natural rewards [18, 20]. Others have shown that in the ventrolateral striatum, both MSN subpopulations are active during specific stages of a motivation task [19].

Our hypothesis was that both MSNs can drive reward and aversion through differential modulation of downstream target regions, namely the VP and VTA. D1-MSNs preferentially innervate VTA GABAergic neurons [51, 52], which in turn disinhibit local dopaminergic neurons [53, 54], although some reports indicate that they can synapse onto both GABAergic and dopaminergic VTA neurons via selective activation of different GABA receptors, or depending on the subregion of the NAc [55, 56]. Our electrophysiological data appears to support a direct inhibition of VTA GABAergic neurons and a later activation (higher latencies—Supplementary Fig. 9) of dopaminergic neurons induced by D1-MSN brief optogenetic stimulation. These results are in line with the work of Kupchik et al. which has also shown a preferential innervation of VTA GABA neurons by D1-MSNs from the core region [12] (in this study we mostly target core (and part of dorsal medial shell) region). But it is also important to refer that some dopaminergic neurons present inhibitory responses, suggesting a monosynaptic input by D1-MSNs. A recent study has elegantly shown that different NAc shell subregions can preferentially innervate either VTA GABA or DA neurons [57], emphasizing the need to perform a detailed and systematic anatomical and electrophysiological characterization of NAc-VTA inputs.

In addition, D1-MSNs stimulation also inhibited 87% of recorded VP neurons, more than the previoulsy 50% reported using patch in slices [12], which is probably explained by technical differences between the two electrophysiological apaproaches. Nevertheless, the observed inhibiton of the VP to VTA tonic inhibitory input can also partially contribute for the observed increase in VTA dopaminergic activity.

Regarding D2-MSNs, brief stimulation inhibited VP activity, disinhibiting VTA dopaminergic activity, in accordance with VP innervation to VTA dopaminergic neurons [30, 32, 58]. In line with this, a recent pharma-optogenetic study from our team showed that the increase in motivation due to D2-MSNs stimulation is dependent on VTA dopaminergic tone and consequent D1R and D2R activation in the NAc [20]. Though the VTA is likely involved, one cannot exclude the contribution of other VP output regions such as the subthalamic nucleus, which has been proposed to play a critical role in ascertaining reward value and magnitude [59], and that can exert an opposite control on cocaine and natural rewards [60].

Regardless of additional mechanisms, one final outcome of both D1- or D2-MSNs brief stimulation was a substantial increase in VTA dopaminergic activity, known to trigger robust behavioral conditioning [23, 61], supporting the place preference of D1- and D2-MSN brief stimulation. In this context, in future studies it will be crucial to evaluate real time dopamine levels in the NAc (and PFC) of stimulated animals.

Contrary, aversion was observed when MSN stimulation was longer (60 s), and in the case of D2-MSNs, it also decreased cocaine conditioning effects. Prolonged D1-MSN activation increased average firing rate of dopaminergic neurons but only during the first second of stimulation. Conversely, VTA GABAergic neurons activity decreased in the first second of stimulation, and then normalized, which was surprising since we predicted a sustained decrease in activity [37]. This suggested that adaptive synaptic mechanisms were occurring, and our hypothesis was that in these conditions, D1-MSNs were coreleasing dynorphin, an endogenous KOR ligand. that has been associated with aversion and negative reinforcement [36, 62]. For example, intra-VTA injection of KOR agonists induces robust conditioned place aversion [62]. Supporting our hypothesis, blocking KOR in the VTA abolished the aversive effect of D1-MSN prolonged stimulation, suggesting that the aversive effect was indeed mediated by dynorphin. Moreover, we observed an initial increase in VTA dopaminergic firing rate, and then the activity quasi normalizes, except for some timepoints, which present subtle activity changes. One hypothesis is that this reflects the net signal of a complex interaction between KOR activation (which decreases activity of dopaminergic neurons in vitro and in vivo [63, 64]), and the reduction in VP inhibitory tone to VTA.

D2-MSN prolonged activation did not cause major electrophysiological differences in VTA GABAergic neurons. In contrast, we observed an increase in dopaminergic firing rate in the first second of stimulation that inverted to a significant decrease in activity afterwards. These electrophysiological findings support the aversive effect observed in the CPP, since it has been shown that reducing VTA dopaminergic activity triggers aversion [65]. In addition, D2-MSN prolonged stimulation reduced cocaine rewarding effects, proposing that the recruitment of this subpopulation may serve as a protective mechanism in drug-exposed individuals [66].

Our in vivo electrophysiological data resembles a very elegant ex vivo study showing that HFS (100 pulses at 100 Hz 20 s) induced LTD at D2-MSNs-to-VP synapses [37]. This effect was mediated by corelease of enkephalin (which only occurs at HFS), that acted presynaptically at DOR in D2-MSNs terminals, decreasing GABA exocytosis [37]. Though our stimulation protocol was different, one hypothesized that it could also induce the corelease of enkephalin, decreasing inhibitory transmission at VP synapses, supporting the observed increase in VP activity after the initial seconds of stimulation. In agreement, we show that intra-VP injection of DOR antagonist abolished the aversive effects of D2-MSN prolonged stimulation (but not in brief).

Optogenetics has been extensively used to dissect neuronal circuits, but it also poses great challenges. It is important to use stimulation protocols that generate a neuronal response closest to the physiological one, which is remarkably difficult, as these are not always characterized. In vivo recordings show that NAc neurons are relatively quiet (<5 Hz), and change firing rate in response to discrete reinforcing stimuli (predictive stimuli or reward itself) in <1 s (or during very few seconds) [67, 68]. Considering this, one could argue that prolonged stimulation could lead to artificial effects not (usually) observed in natural conditions. Yet, in the context of cocaine self-administration, prolonged excitation/inhibition of a fraction of NAc neurons can also occur [69, 70]. So, we believe that it is crucial to perform additional studies evaluating the activity pattern of MSNs in freely moving animals during different rewarding/aversive tasks in order to better understand how the two subpopulations work to generate behavior.

This study also highlights the importance of testing different stimulation/inhibition parameters and evaluating their impact not only in the manipulated region, but also in downstream targets and in behaviour. Although the increases in firing rate of NAc neurons of brief and prolonged stimulation are similar to those found in freely moving animals in response to natural rewards [67, 68, 71], the evoked effects in the VTA and VP were clearly different between the two stimuli.

In this work, we contributed to explain contradictory results in the field, by showing that optogenetic stimulation of NAc D1- or D2-MSNs can drive both reward and aversion, depending on their stimulation pattern. In addition, we show that cocaine conditioning is also differentially affected by manipulation of these NAc subpopulations. This work highlights that the striatal functional organization is more complex than classically proposed, and that additional studies are needed to disentangle the contribution of each subpopulation in valenced behaviors, which is of utmost importance to better comprehend neuropsychiatric disorders such as depression and addiction, that present marked striatal dysfunction [43–45, 47, 48].

## Supplementary information

Supplementary Information

Supplementary Figures
