# Supplementary material for: Nucleus accumbens medium spiny neurons subtypes signal both reward and aversion
Source: Mol Psychiatry. 2019 Aug 28;25(12):3241–55. doi: 10.1038/s41380-019-0484-3 (PMC7714688; doi:10.1038/s41380-019-0484-3)
Supplement: Supplementary file 1 — Supplementary Information [file 41380_2019_484_MOESM1_ESM.pdf]

Supplementary Information – Soares-Cunha *et al*.

Materials and Methods

Animals

C57/Bl6 transgenic mice (2 months old) were housed in groups of 3-5 animals. The following heterozygous mouse lines were used: D1-cre (line EY262, Gensat.org) and D2-cre (line ER44, Gensat.org). A mating scheme of male heterozygous x female wild-type was used.

Genotyping

DNA was isolated from tail biopsy using the Citogene DNA isolation kit (Citomed, Lisbon, Portugal). In a single PCR genotyping tube, the primers Drd1a F1 (5'-GCTATGGAGATGCTCCTGATGGAA-3') and CreGS R1 (5'-CGGCAAACGGACAGAAGCATT-3') were used to amplify the D1-cre transgene (340 bp), and the primers Drd2 (32108) F1 (5'-GTGCGTCAGCATTTGGAGCA-3') and CreGS R1 (5'-CGGCAAACGGACAGAAGCAT-3') to amplify the D2-cre transgene (700 bp). An internal control gene (lipocalin 2, 500 bp) was used in the PCR (LCN_1 (5'-GTCCTTCTCACTTTGACAGAAGTCAGG-3') and LCN_2 (5'-CACATCTCATGCTGCTCAGATAGCCAC-3'). Heterozygous mice were discriminated from the wild-type mice by the presence of two amplified DNA products corresponding to the transgene and the internal control gene. Gels were visualized with GEL DOC EZ imager (Bio-Rad, Hercules, CA, USA) and analyzed with the Image Lab 4.1 (Bio-Rad, Hercules, CA, USA).

Surgery and cannula implantation

Mice were anaesthetized with 75 mg kg^{-1} ketamine (Imalgene, Merial, Lyon, France) plus 1mg kg^{-1} medetomidine (Dorbene, Cymedica, Horovice, Czech Republic). Virus (500nl) was unilaterally injected into the NAc- coordinates from bregma[30]: +1.3mm anteroposterior (AP), +0.9mm mediolateral (ML), and -4.0mm dorsoventral (DV); using an 30-gauge needle

Hamilton syringe (Hamilton Company, Reno, NV, USA), at a rate of 100nl min^{-1}. After injection, the syringe was left in place for 5 minutes to allow diffusion. Mice were then implanted with an optic fiber (200 µm core fiber optic; Thorlabs, Newton, NJ, USA) with 2.5mm stainless steel ferrule (Thorlabs, Newton, NJ, USA) using the injection coordinates (with the exception of DV: -3.9 mm) that were secured to the skull using dental cement (C&B kit, Sun Medical, Shiga, Japan). Mice were removed from the stereotaxic frame, sutured and let to recover for two weeks before initiation of the behavioral protocols. All animals were treated 30 minutes before surgery and 6 hours after surgery with an analgesic – buprenorphine at 0.05 mg kg^{-1} (Bupaq, Richter Pharma, Wels, Austria).

For optical stimulation+drug delivery in terminals, virus (500nl) was unilaterally injected into the NAc of D1- or D2-cre mice (coordinates from bregma[29]: +1.3mm anteroposterior (AP), +0.9mm mediolateral (ML), -4.0mm dorsoventral (DV)), and a guide cannula (Plastics 1, Roanoke, VA, USA) was implanted in the VTA (coordinates from bregma[29]: -3.2mm AP, +0.5mm ML, and 4.5mm DV) and VP (coordinates from bregma[29]: 0.1mm AP, +1.6mm ML, and -3.9mm DV), respectively.

Drugs

Drugs (Naltrindole 0.1µg or norbinaltorphimine 1µg) or vehicle were delivered 20 minutes before animals performed the CPP test, through a fluid system chronically implanted in the VP or VTA. Injections were performed using a 5µl gastight seringe (Hamilton), attached to the implanted injection cannula of the rats through 22-gauge tubing, at a constant rate of 0.5µl/min.

Optical stimulation

For all optogenetic experiments using ChR2, 5mW of blue light (at the tip of the fiberoptic) was generated by 473nm DPSS laser (CNI Laser, Changchun, China) and unilaterally delivered to mice through fiberoptic patch cords (0.22NA, 200µm diameter; Thorlabs, Newton, NJ, USA) that were attached to the implanted ferrule. For optogenetic

experiments using eNpHR, 5mW of yellow light (at the tip of the fiberoptic) was generated by 589nm DPSS laser (CNI Laser, Changchun, China) and unilaterally delivered as above. Laser output was controlled using a pulse generator (Master-8; AMPI, New Ulm, MN, USA) to deliver light.

Conditioned Place Preference – CPP – with optical stimulation

The CPP apparatus consisted of two compartments with different patterns on floors and walls, separated by a neutral area (Med Associates Inc., St. Albans, VT, USA); location was monitored using a computerized photo-beam system (Med Associates Inc., St. Albans, VT, USA). The CPP test consisted of three phases over 4 days (according to previous description[31]. Animals were exposed to 1 pre-test day (15 minutes) and 2 conditioning days (30 minutes; 1 day with and 1 day without optical stimulation), and a post-test day (15 minutes). Results are expressed as the difference of time spent in the stimulus-paired chamber and the no-stimulus-paired chamber on the post-test day, and total time spent on the stimulus and no stimulus-paired chamber on the post-test day.

Optical stimulation: (1) brief: 1s, 473 nm; frequency of 40 Hz; light pulses of 12.5 ms duration, every minute; (2) prolonged: 60s, 473 nm; frequency of 40 Hz; light pulses of 12.5 ms, with an interval of 1 minute. Optical inhibition: 589 nm; constant light delivered over 10 seconds, every minute.

Other optical stimulation protocols: (3) brief: 1s, 473 nm; frequency of 20 Hz; light pulses of 25 ms duration, every minute; (4) prolonged: 60s, 473 nm; frequency of 20 Hz; light pulses of 25 ms, with an interval of 1 minute; (5) brief: 1s, 473 nm; frequency of 10 Hz; light pulses of 50 ms duration, every minute; (4) prolonged: 60s, 473 nm; frequency of 10 Hz; light pulses of 50 ms, with an interval of 1 minute.

Conditioned Place preference with cocaine + optical stimulation

The CPP apparatus consisted of two compartments with different patterns on floors and walls, separated by a neutral area (Med Associates Inc., St. Albans, VT, USA); location was

monitored using a computerized photo-beam system (Med Associates Inc., St. Albans, VT, USA).

Animals were exposed to 1 pre-test day (20 minutes) and 2 conditioning days [with 2 daily sessions (30 minutes)] and a post-test day (20 minutes). On conditioning days, optic fibers were secured to the cannula prior to saline or cocaine i.p. injections. Mice were conditioned to saline and no light for a 30-min session and cocaine (5 mg/kg) and blue light for a 30-min session over two days.

Results are expressed as the difference of time spent in the cocaine + stimulus-paired chamber and the saline + no-stimulus-paired chamber on the post-test day, and total time spent on the cocaine + stimulus and saline + no stimulus-paired chamber on the post-test day.

Optical stimulation: (1) brief: 1s, 473 nm; frequency of 40 Hz; light pulses of 12.5 ms duration, every minute; (2) prolonged: 60s, 473 nm; frequency of 40 Hz; light pulses of 12.5 ms, with an interval of 1 minute. Optical inhibition: 589 nm; constant light delivered over 10 seconds, every minute.

Real-Time Place Preference – RTPP

Briefly, mice were tested in a custom-made black plastic arena (60 x 60 x 40 cm) consisting of two indistinguishable chambers, for 15 min. One chamber was paired with light stimulation (40 Hz, 12.5 ms light pulses) and the other was paired with no stimulation. The choice of paired chamber was counterbalanced across mice. Results are presented as total time spent in each chamber.

Locomotor Activity

Locomotor activity was evaluated as previously described[24], in an open field arena (43.2 cm x 43.2 cm) with transparent acrylic walls and white floor (Med Associates Inc., St. Albans, VT, USA). Briefly, mice were attached to an optical fiber connected to a laser (473 nm or 589 nm) and immediately placed in the center of the arena. Locomotion was monitored online

over a period of 30 minutes (stimulation was given similarly as in the CPP conditioning session). Distance traveled during the 30-minute session was used as indicator of locomotor activity.

To assess cocaine-induced locomotor effects mice were injected with cocaine (5mg/kg, i.p.) and immediately placed in the open field apparatus, where locomotion was monitored for 30 minutes.

In vivo single cell electrophysiology

Four weeks after injection of the virus, animals were submitted to a stereotaxic surgery for the placement of the optic fiber and recording electrodes. Mice were anesthetized with urethane (1.75 g Kg^{-1}, Sigma now Merck KGaA, Darmstadt, Germany).

A recording electrode coupled with a fiber optic patch cable (Thorlabs, Newton, NJ, USA) was placed in the NAc (coordinates from bregma: +1.3 mm AP, +0.9 mm ML, and 3.5 to 4.2 mm DV), using a stereotaxic frame (David Kopf Instruments, Tujunga, CA, USA) with non-traumatic ear bars (Stoeling, Wood Dale, IL, USA). Other recording electrodes were placed in the VP (coordinates from bregma: -0.12mm AP, +1.6mm ML, and -3.5 to 4mm DV) and in the VTA (coordinates from bregma: -3.2mm AP, +0.5mm ML, and 4 to 4.8mm DV). Single neuron activity was recorded extracellularly with a tungsten electrode (tip impedance 5-10 Mat 1 kHz). Recordings were amplified and filtered by the Neurolog amplifier (NL900D, Digitimer Ltd, Hertfordshire, UK) (low-pass filter at 500 Hz and high-pass filter at 5 kHz). Spontaneous activity of single neurons was recorded to establish baseline for at least 60 seconds. Stimulation was performed by intracranial light delivery, through a DPSS 473 nm laser system (CNI Laser, Changchun, China), controlled by a stimulator (Master-8, AMPI, New Ulm, MN, USA). Stimulation protocols: (1) brief: 1s, frequency of 40 Hz, light pulses of 12.5 ms duration, 5 mW at the tip of the fiber; (2) prolonged: 60s, frequency of 40 Hz, light pulses of 12.5 ms duration, 5 mW at the tip of the fiber. Spikes of single neurons were discriminated and data sampling was performed using a CED Micro1401 interface and Spike 2 software (Cambridge Electronic Design, Cambridge, UK).

Firing rate stimulus histograms were calculated for the baseline (60 s prior to stimulation, using a bin size of 1s), stimulation period and after stimulation period (60 s after the end of stimulation, using a bin size of 1s). An additional set of animals (4 animals/genotype/brain region) was recorded for a 30minute period with the same number of optical stimulations as in the CPP test (Supplementary Figures 7-8).

We defined the neuronal instantaneous firing rate of the *i*-th neuron as given by r_i (a_k, b_k) = h (u_i, a_k, b_k, w), where h is a histogram function over the vector u_i which stores the spiking times of the *i*-th neuron in the population, within the time interval [a_k, b_k), and w was the bin size for h (usually w=1s). In order to calculate the PETH, each recorded spike train from a single neuron was aligned by the onset of optical stimulation. For each neuronal instantaneous firing rate r_i the average activity during baseline was subtracted (r_i=r_i-avg(r_i[t<40s]), and then neurons were sorted by the average activity during optical stimulation.

Spike latency was determined as the time (in milliseconds (ms)) elapsed from the beginning of optical stimulation and the first spike detected after beginning of such stimulus, for each recorded neuron (regardless if that neuron is inhibited, excited or presenting no change in activity from baseline).

NAc neurons were classified according to previous descriptions[27,34]. In short, fast-spiking interneurons – putative parvalbumin-containing neurons (pFSs) – were identified has having a waveform half-width of less that 100μs and a baseline firing rate higher that 10Hz; tonically active putative cholinergic interneurons (pCINs) were identified as those with a waveform half-width bigger that 300μs. Putative MSNs (pMSNs) were identified as those with baseline firing rate lower that 5Hz and that do not met the waveform criteria for pCIN or pFS neurons. NAc MSNs were further separated in pMSN A, neurons which activity increased >20% from baseline during stimulation, or pMSN B, which activity did not present this change during the stimulation period [24,33]. A similar separation was performed for pCINs and pFS.

VP GABAergic neurons were identified as those having a baseline firing rate between 0.2 Hz and 18.7 Hz[35]. Other non-identified neurons (corresponding to less that 5% of

recorded cells) were excluded from the analysis.

Single units in the VTA were separated into those putative dopaminergic (pDAergic) and putative GABAergic (pGABAergic). This classification was based on firing rate and waveform duration[36–38]. Cells presenting baseline firing rate lower that 10Hz and a waveform duration higher than 1.5ms were considered pDAergic neurons. Cells presenting baseline firing rate higher than 10Hz and waveform duration lower than 1.5ms were classified as pGABAergic. Other single units that did not fit in any classification (less that 5% of recorded cells) were excluded from the analysis.

Immunofluorescence (IF)

In brief, mice were deeply anesthetized and then transcardially perfused with 0.9% saline followed by 4% paraformaldehyde (PFA). Brains were removed, post-fixed in 4% PFA and sectioned in a vibratome. Sections treated with citrate buffer, blocked, and then incubated with the primary antibodies mouse anti-D2 receptor (B-10) (1:400, sc-5303, Santa Cruz Biotechnology, Dallas, TX, USA), and goat anti-GFP (1:500, ab6673, Abcam, Cambridge, UK), or mouse anti-D1 receptor (1:200, NB110-60017, Novus, Littleton, CO, USA) and goat anti-GFP (1:500, ab6673, Abcam, Cambridge, UK). Appropriate secondary fluorescent antibodies were used (1:500, Invitrogen, Carlsbad, CA, USA).

Cell density estimation was obtained by normalizing positive cells with the corresponding area, determined using a confocal microscope (Olympus FluoViewTMFV1000, Olympus, Tokyo, Japan) and the StereoInvestigator software (Microbrightfield). For each animal, 5 slices containing the NAc were photographed.

Statistical analysis

Prior to any statistical comparison between groups, normality tests (Shapiro-Wilks (S-W)) were performed for all data analysed. When normality assumptions were met, statistical analysis using parametric tests was performed: comparison between two groups in the behavioural parameters was made using Student's *t*-test (when normality assumptions were

not met Mann-Whitney was performed instead); comparison between behaviour on the ON side and the OFF side within the same subject was performed using paired t-test; Analysis of Variance (ANOVA) for repeated measures was used to compare firing rate before, during and after stimulation, and Bonferroni's *post hoc* multiple comparisons was used for group differences determination (when normality assumptions were not met Friedman's test was performed, and Dunn's multiple comparison for post hoc analysis).

For the analysis of electrophysiological temporal variation, for each time bin, the activity during stimulation was considered significant when on that time bin the activity was out 95% of the distribution of the baseline activity. In the case of brief optogenetic stimulation datasets, given the zero mean baseline activity, the p-value was calculated as the fraction of samples in baseline activity, which were, in absolute value, greater than the value of the onset activity (single sample). For prolonged stimulation datasets, Komlogorov-Smirnov for 2 samples was performed to determine differences between the distribution of the stimulus period and the baseline.

All statistical analysis was performed using Python packages (numpy 1.10.1; scipy 0.18.1, Python Software Foundation, Beaverton, OR, USA) and GraphPad (Prism 7, La Jolla, CA, USA).

Results are presented as mean ± SEM. All of the statistical details of experiments can be found throughout the results description; these include the statistical tests used and exact p-value. The n for each experiment is indicated in the figures' legends.

Supplementary Table 1. Periods of changes in neuronal activity of VP and VTA neurons in comparison to baseline during prolonged optical stimulation of D1-MSNs. = refers to no change from baseline, ↑ and ↓ refers to increase and decrease of at least 20% from baseline ($p<0.05$), respectively.

**D1- MSN prolonged (60s) optical stimulation**			
**# Stimulus**	**VP**	**VTA (DAergic)**	**VTA (GABAergic)**
**1**	↑ 1-2" ; = 2-60"	↑ 1" ; ↓ 2-60"	↓ 1-2" ; = 2-60"
**2**	↑ 1-2" ; = 2-60"	↑ 1" ; ↓ 2-60"	↓ 1-2" ; = 2-60"
**3**	↑ 1-2" ; = 2-60"	↑ 1" ; ↓ 2-60"	↓ 1-2" ; = 2-60"
**4**	↑ 1-2" ; = 2-60"	↑ 1" ; ↓ 3-60"	↓ 1-3" ; = 3-60"
**5**	↑ 1-2" ; = 2-60"	↑ 1" ; ↓ 2-60"	↓ 1-2" ; = 2-60"
**6**	↑ 1-2" ; = 2-60"	↑ 1" ; ↓ 2-60"	↓ 1-2" ; = 2-60"
**7**	↑ 1-3" ; = 3-60"	↑ 1" ; ↓ 3-60"	↓ 1-2" ; = 2-60"
**8**	↑ 1-3" ; = 3-60"	↑ 1" ; ↓ 3-60"	↓ 1-2" ; = 2-60"
**9**	↑ 1-3" ; = 3-60"	↑ 1" ; ↓ 3-60"	↓ 1-2" ; = 2-60"
**10**	↑ 1-2" ; = 2-60"	↑ 1" ; ↓ 3-60"	↓ 1-2" ; = 2-60"
**11**	↑ 1-3" ; = 3-60"	↑ 1" ; ↓ 3-60"	↓ 1-2" ; = 2-60"
**12**	↑ 1-2" ; = 2-60"	↑ 1" ; ↓ 3-60"	↓ 1-3" ; = 3-60"
**13**	↑ 1-2" ; = 2-60"	↑ 1" ; ↓ 3-60"	↓ 1-2" ; = 2-60"
**14**	↑ 1-2" ; = 2-60"	↑ 1" ; ↓ 3-60"	↓ 1-3" ; = 3-60"
**15**	↑ 1-2" ; = 2-60"	↑ 1" ; ↓ 3-60"	↓ 1-2" ; = 2-60"

Supplementary Table 2. Periods of changes in neuronal activity of VP and VTA neurons in comparison to baseline during prolonged optical stimulation of D2-MSNs. = refers to no change from baseline, ↑ and ↓ refers to increase and decrease of at least 20% from baseline ($p<0.05$), respectively.

**D2- MSN prolonged (60s) optical stimulation**			
**# Stimulus**	**VP**	**VTA (DAergic)**	**VTA (GABAergic)**
**1**	↓ 1-2” ; ↑ 2-60”	↑ 1-2” ; ↓ 2-60”	= 1-60”
**2**	↓ 1-2” ; ↑ 2-60”	↑ 1-2” ; ↓ 3-60”	= 1-60”
**3**	↓ 1-2” ; ↑ 2-60”	↑ 1-2” ; ↓ 3-60”	= 1-60”
**4**	↓ 1-2” ; ↑ 2-60”	↑ 1-2” ; ↓ 3-60”	= 1-60”
**5**	↓ 1-3” ; ↑ 3-60”	↑ 1-2” ; ↓ 3-60”	= 1-60”
**6**	↓ 1-2” ; ↑ 2-60”	↑ 1-2” ; ↓ 3-60”	= 1-60”
**7**	↓ 1-2” ; ↑ 2-60”	↑ 1-2” ; ↓ 3-60”	= 1-60”
**8**	↓ 1-3” ; ↑ 3-60”	↑ 1-2” ; ↓ 2-60”	= 1-60”
**9**	↓ 1-3” ; ↑ 3-60”	↑ 1-2” ; ↓ 3-60”	= 1-60”
**10**	↓ 1-3” ; ↑ 3-60”	↑ 1-2” ; ↓ 2-60”	= 1-60”
**11**	↓ 1-3” ; ↑ 3-60”	↑ 1-2” ; ↓ 3-60”	= 1-60”
**12**	↓ 1-3” ; ↑ 3-60”	↑ 1-2” ; ↓ 3-60”	= 1-60”
**13**	↓ 1-3” ; ↑ 3-60”	↑ 1-2” ; ↓ 3-60”	= 1-60”
**14**	↓ 1-2” ; ↑ 2-60”	↑ 1-2” ; ↓ 2-60”	= 1-60”
**15**	↓ 1-3” ; ↑ 3-60”	↑ 1-2” ; ↓ 3-60”	= 1-60”
