# Supplementary material for: Nucleus accumbens medium spiny neurons subtypes signal both reward and aversion
Source: Mol Psychiatry. 2019 Aug 28;25(12):3241–55. doi: 10.1038/s41380-019-0484-3 (PMC7714688; doi:10.1038/s41380-019-0484-3)
Supplement: Supplementary file 2 — Supplementary Figures [file 41380_2019_484_MOESM2_ESM.pdf]

Supplementary Figure 1

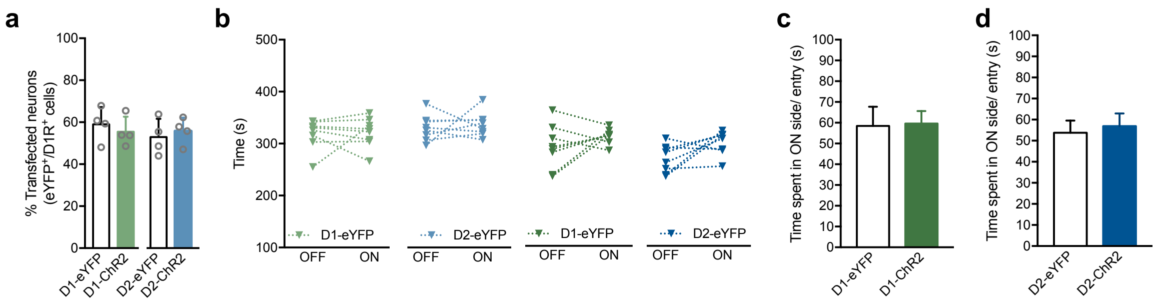

Supplementary Figure 1. Brief and prolonged D1- and D2-MSN stimulation oppositely regulate CPP. (**a**) Quantification of immunofluorescence for dopamine receptor D1 or D2 and YFP. Around 60% of neurons were transfected with ChR2 or eYFP (n=4 animals). (**b**) Total time spent in the stimulus-associated chamber (ON) and the no-stimulus-associates chamber (OFF) by D1-eYFP and D2-eYFP mice during the post-test session of the CPP with brief optical stimulation (left, light colors) and total time spent in the stimulus-associated chamber (ON) and the no-stimulus-associates chamber (OFF) by D1-eYFP and D2-eYFP mice during the post-test session of the CPP with prolonged optical stimulation (right, dark colors). (**c-d**) Time spent in each entry in the RTPP test. ($n_{D1\text{-}eYFP}$=7; $n_{D2\text{-}eYFP}$=8).

Supplementary Figure 2

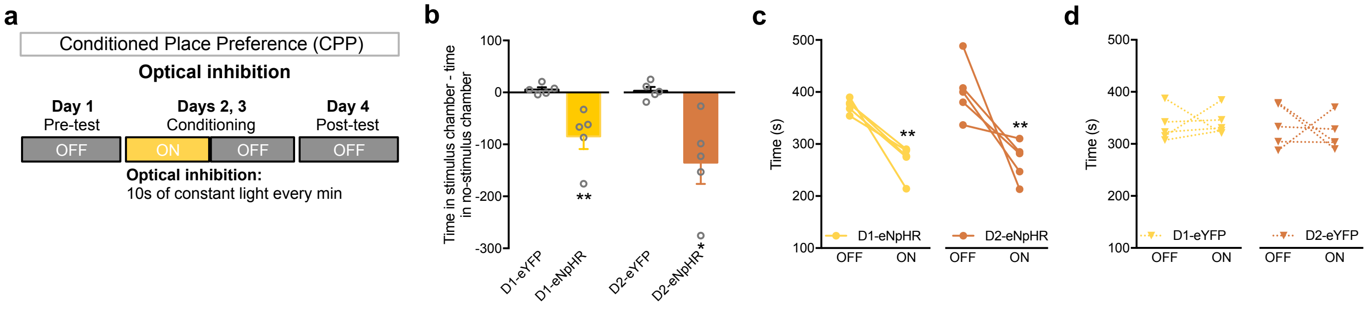

Supplementary Figure 2. Optogenetic inhibition of D1- or D2-MSNs induces aversion. (**a**) Schematic representation of optogenetic inhibition parameters in the CPP test. (**b**) Optical inhibition of D1- or D2-MSNs causes significant aversion to the stimulus-associated chamber ($n_{D1\text{-}eYFP}$=5, $n_{D1\text{-}eNpHR}$=5; $n_{D2\text{-}eYFP}$=5, $n_{D2\text{-}eNpHR}$=5). (**c**) All D1-eNpHR and D2-eNpHR mice spend less time in the ON side. (**d**) No differences were found between time spent in the ON side and the OFF side of the CPP apparatus of both D1-eYFP and D2-eYFP mice. *p<0.05, **p<0.01. Data is represented as mean ± SEM.

Supplementary Figure 3

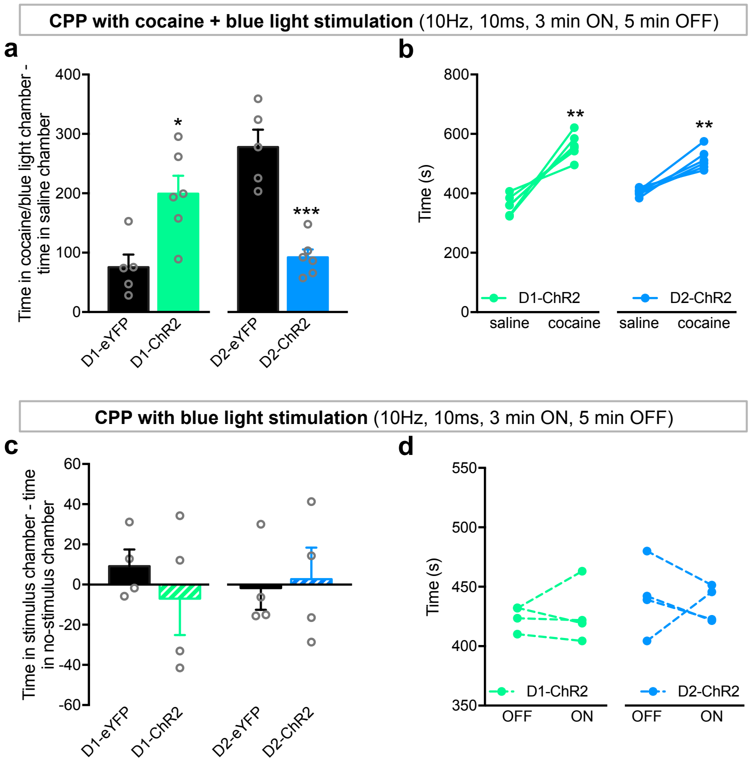

Supplementary Figure 3. Replication of the CPP protocol described by Lobo *et al*, 2010. Optogenetic activation (10Hz, 10ms, 3 min ON, 5 min OFF) of D1- or D2-MSNs has no effect itself, and oppositely regulates cocaine reward. (**a-b**) Optical activation of D1-MSNs enhances cocaine (5 mg/kg) conditioning in D1-ChR2 mice when compared with D1-eYFP controls ($n_{D1\text{-}eYFP}$=5, $n_{D1\text{-}ChR2}$=6; *Student's t test*, t_9=3.2, p=0.01). In contrast, optical activation of D2-MSNs decreased cocaine (5 mg/kg) conditioning in D2-ChR2 mice when compared with D2-eYFP controls ($n_{D2\text{-}eYFP}$=5, $n_{D2\text{-}ChR2}$=6; *Student's t test*, t_9=6.2, p=0.0002). (**c,d**) No preference was observed for D1- or D2-MSN stimulation in the absence of cocaine ($n_{D1\text{-}eYFP}$=4, $n_{D1\text{-}ChR2}$=4; $n_{D2\text{-}eYFP}$=4, $n_{D2\text{-}ChR2}$=4). *p<0.05, **p<0.01, ***p<0.001. Data is represented as mean ± SEM.

Supplementary Figure 4

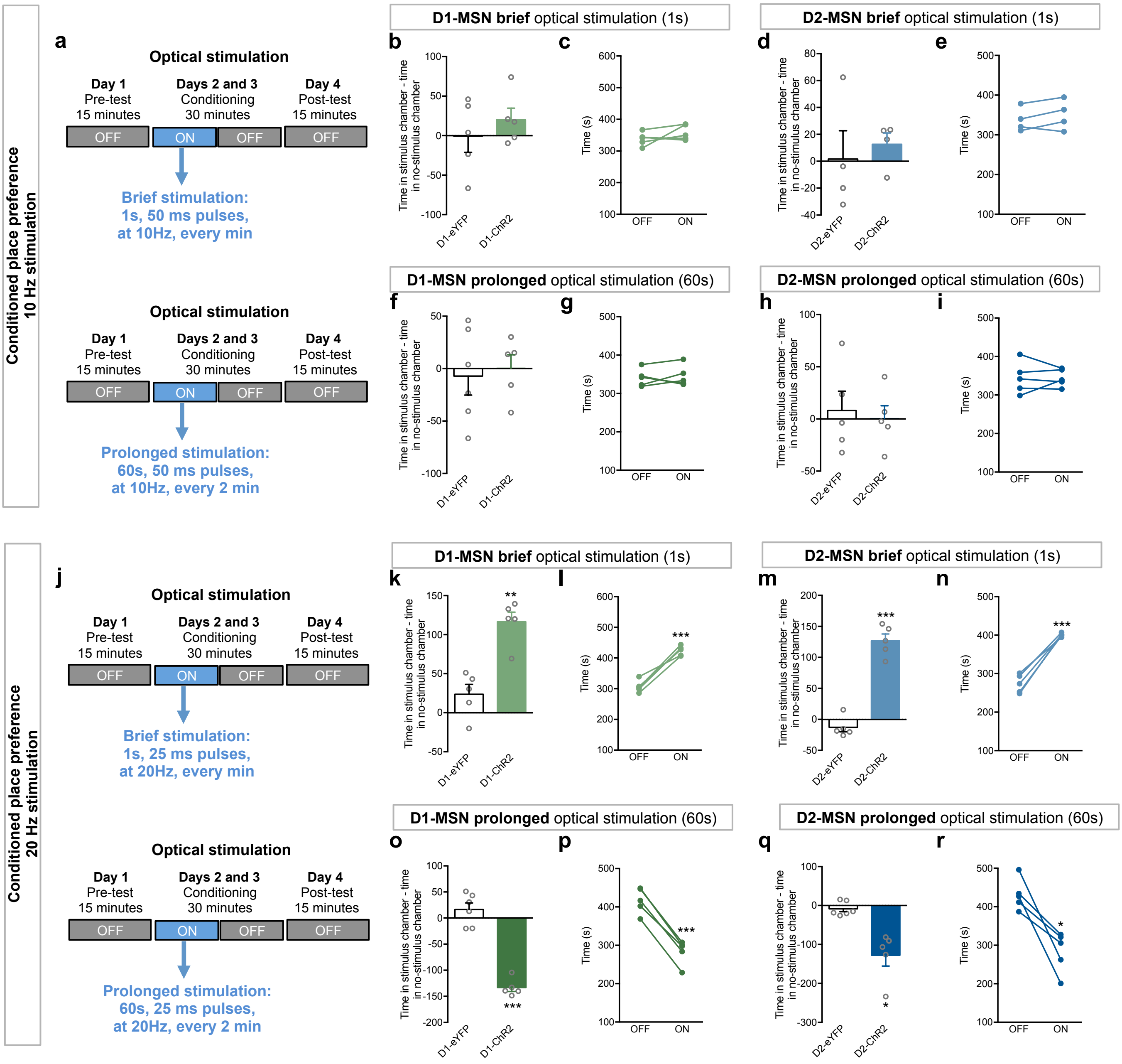

Supplementary Figure 4. Behavioral effect of different protocols of D1- and D2-MSN stimulation. (**a**) Schematic representation of brief and prolonged optogenetic stimulation in the CPP test, using 10Hz frequency. (**b-c**) Brief (10Hz, 1s) optical stimulation of NAc D1-MSNs did not induce preference nor aversion ($n_{D1\text{-}eYFP}$=5, $n_{D1\text{-}ChR2}$=5; *Mann-Whitney test*, U=10.0, p=0.6905). (**d-e**) Brief (10Hz, 1s) optical stimulation of NAc D2-MSNs did not induce preference ($n_{D2\text{-}eYFP}$=4, $n_{D2\text{-}ChR2}$=4; *Student's t test*, t_6=0.5, p=0.6467). (**f-g**) Prolonged (10Hz, 60s) optical stimulation of NAc D1-MSNs does not induce place preference or aversion ($n_{D1\text{-}eYFP}$=6, $n_{D1\text{-}ChR2}$=5; t_9=0.3, p=0.7565). (**h-i**) Prolonged (10Hz, 60s) optical stimulation of NAc D2-MSNs does not induce place preference or aversion ($n_{D2\text{-}eYFP}$=5, $n_{D2\text{-}ChR2}$=5; t_8=0.3, p=7395). (**j**) Schematic representation of brief and prolonged optogenetic stimulation in the CPP test, using 20Hz frequency. (**k-l**) Brief (20Hz, 1s) optical stimulation of NAc D1-MSNs induces place preference ($n_{D1\text{-}eYFP}$=5, $n_{D1\text{-}ChR2}$=5; *Mann-Whitney test*, U=0.0, p=0.0079; l- *Paired t test*, t_4=9.4, p=0.0007). (**m-n**) Brief (20Hz, 1s) optical stimulation of NAc D2-MSNs induces place preference ($n_{D2\text{-}eYFP}$=5, $n_{D2\text{-}ChR2}$=5; *Student's t test*, t_8=10.6, p<0.0000; n- *Paired t test*, t_4=11.5, p=0.0003). (**o-p**) Prolonged (20Hz, 60s) optical stimulation of NAc D1-MSNs induces place aversion ($n_{D1\text{-}eYFP}$=6, $n_{D1\text{-}ChR2}$=5; t_9=9.6, p<0.0000; p- *Paired t test*, t_4=17.5, p<0.0000). (**q-r**) Prolonged (20Hz, 60s) optical stimulation of NAc D2-MSNs induces place aversion ($n_{D2\text{-}eYFP}$=6, $n_{D2\text{-}ChR2}$=5; t_9=4.6, p=0.0014; r- *Paired t test*, t_4=4.4, p=0.0120). *p<0.05, **p<0.01, ***p<0.001. Data is represented as mean ± SEM.

Supplementary Figure 5

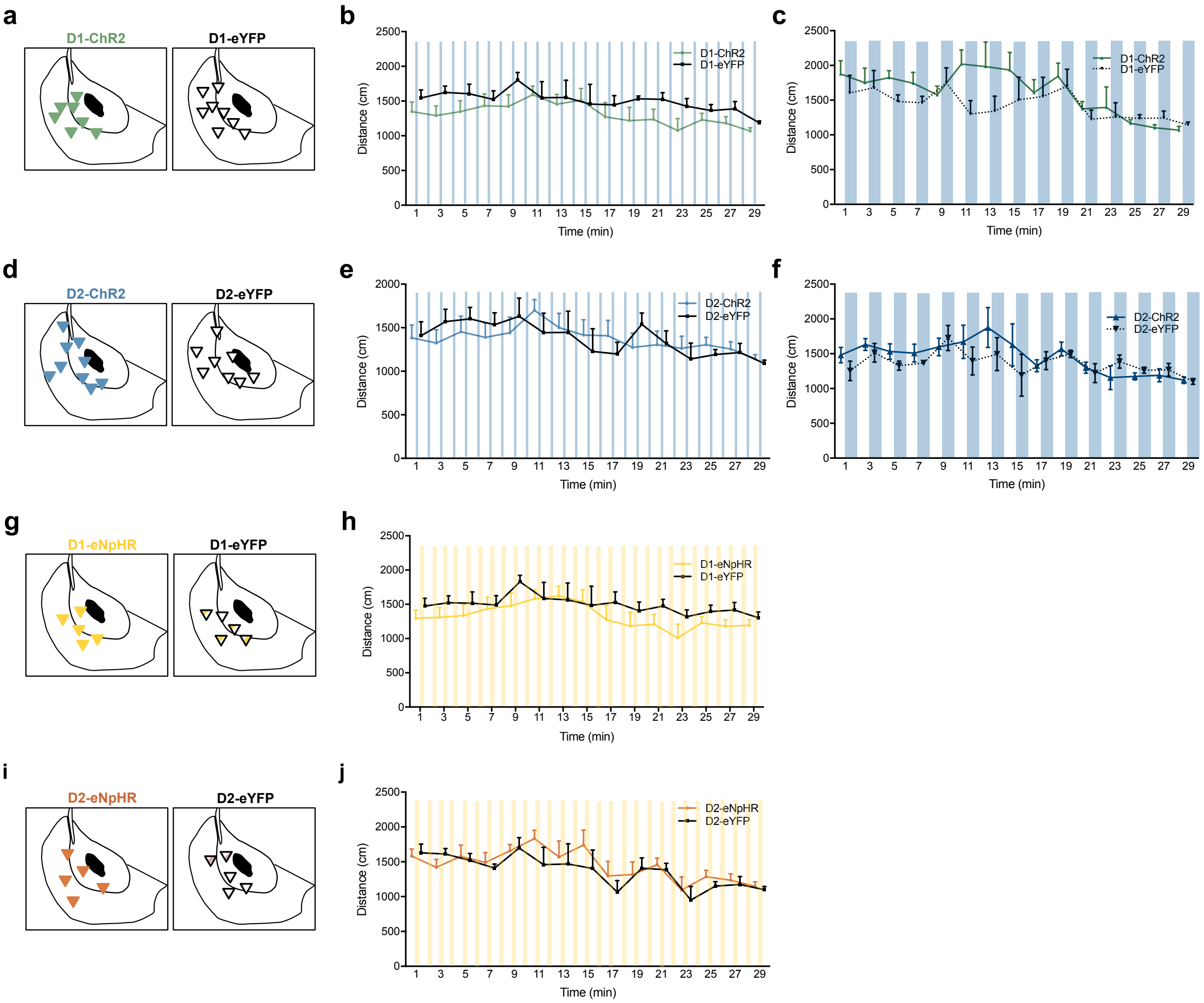

Supplementary Figure 5. Histological representation of optic fiber placement and locomotor activity of D1-cre and D2-cre mice. Optic fiber placement of (**a**) D1-ChR2 and D1-eYFP mice ($n_{D1\text{-}ChR2}$=7, n_{eYFP}=8), (**d**) D2-ChR2 and D2-eYFP mice ($n_{D2\text{-}ChR2}$=9, n_{eYFP}=8), (**g**) D1-eNpHR and D1-eYFP ($n_{D1\text{-}eNpHR}$=5, n_{eYFP}=5), (**i**) D2-eNpHR and D1-eYFP ($n_{D2\text{-}eNpHR}$=5, n_{eYFP}=5). No differences in locomotor activity of stimulated animals were found (**b, c, e, f, h, j**). Data are represented as mean ± SEM. Blue stripes correspond to optogenetic stimulus.

Supplementary Figure 6

a

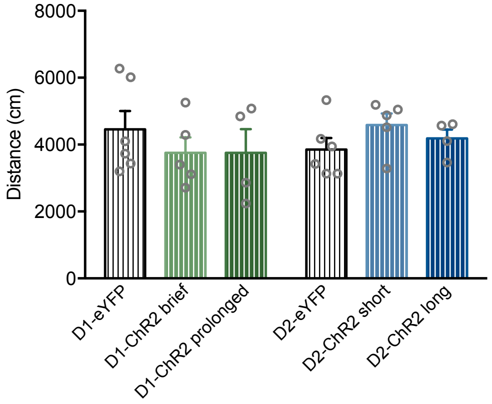

Supplementary Figure 6. Locomotion in cocaine-induced CPP test. No significant chances in locomotion caused by cocaine exposure in D1-cre and D2-cre optically stimulated mice. Data are represented as mean ± SEM.

Supplementary Figure 7

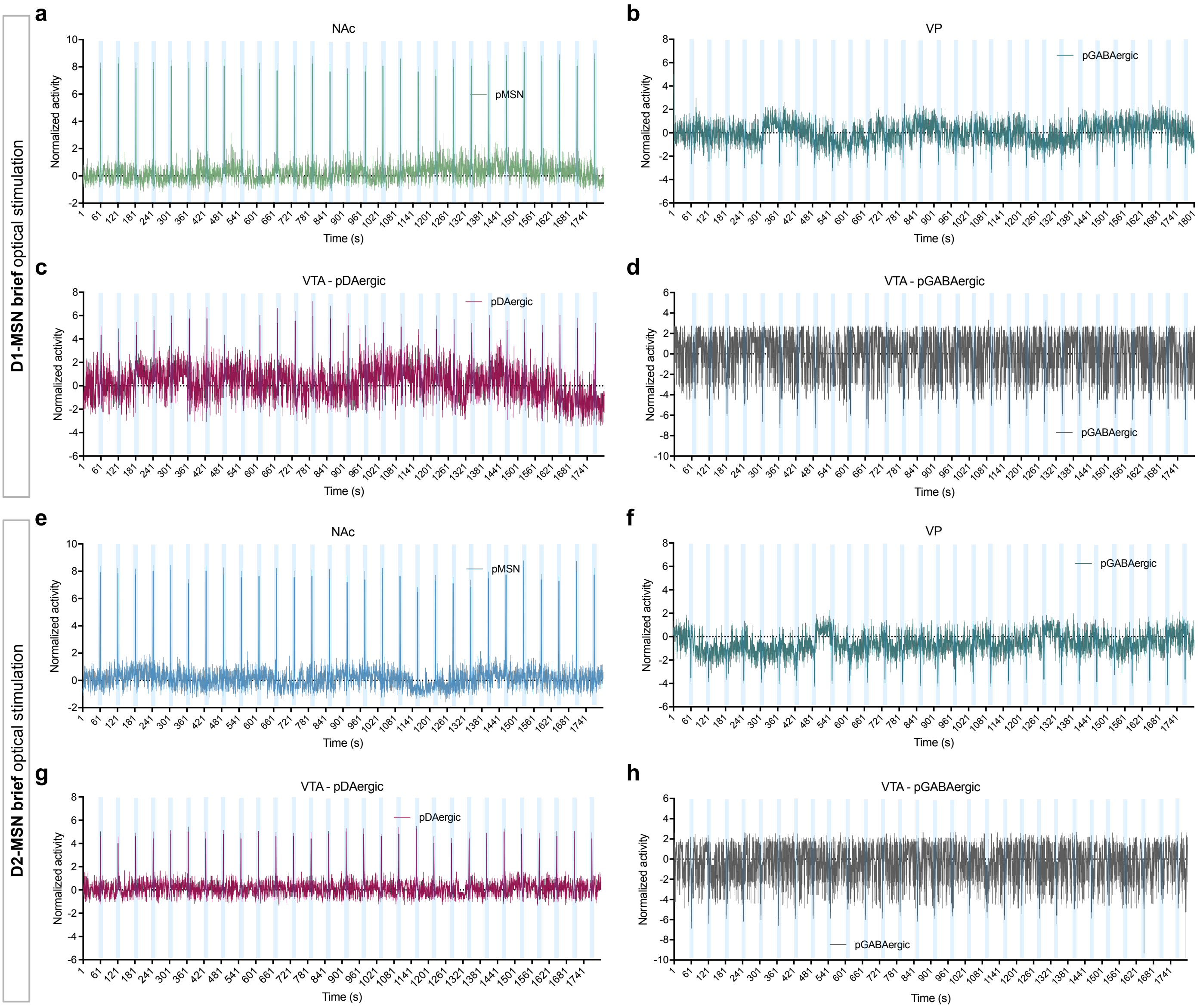

Supplementary Figure 7. Electrophysiological responses of NAc, VP and VTA during 30 minute protocol of brief stimulation. A 30 minutes session with 1s stimulation (12.5 ms pulses at 40 Hz) every min (which is equivalent to CPP test) was recorded, in order to evaluate the neuronal responses to repetitive stimulations. (**a**) Temporal variation of NAc pMSNs activity with D1-MSN brief stimulation; note the increase in activity during optical stimulation (blue; *stim vs no stim*, p<0.0000, n=12 cells/6mice). (**b**) Temporal variation of VP pGABAergic neuronal activity with D1-MSN brief stimulation; note the decrease in activity during optical stimulation (blue; *stim vs no stim*, p<0.0000, n=9 cells/5mice). (**c**) Temporal variation of VTA pDAergic neuronal activity with D1-MSN brief stimulation; note the increase in activity during optical stimulation (blue; *stim vs no stim*, p<0.0000, n=6 cells/5mice). (**d**) Temporal variation of VTA pGABAergic neuronal activity with D1-MSN brief stimulation; note the decrease in activity during optical stimulation (blue; *stim vs no stim*, p<0.0000, n=3 cells/5mice). (**e**) Temporal variation of NAc pMSNs activity with D2-MSN brief stimulation; note the increase in activity during optical stimulation (blue; *stim vs no stim*, p<0.0000, n=12 cells/5mice). (**f**) Temporal variation of VP pGABAergic neuronal activity with D2-MSN brief stimulation; note the decrease in activity during optical stimulation (blue; *stim vs no stim*, p<0.0000, n=10 cells/5mice). (**g**) Temporal variation of VTA pDAergic neuronal activity with D2-MSN brief stimulation; note the increase in activity during optical stimulation (blue; *stim vs no stim*, p<0.0000, n=6 cells/5mice). (**h**) Temporal variation of VTA pGABAergic neuronal activity with D2-MSN brief stimulation; note the decrease in activity during optical stimulation (blue; *stim vs no stim*, p<0.0000, n=3 cells/5mice). Data are represented as mean ± SEM.

Supplementary Figure 8

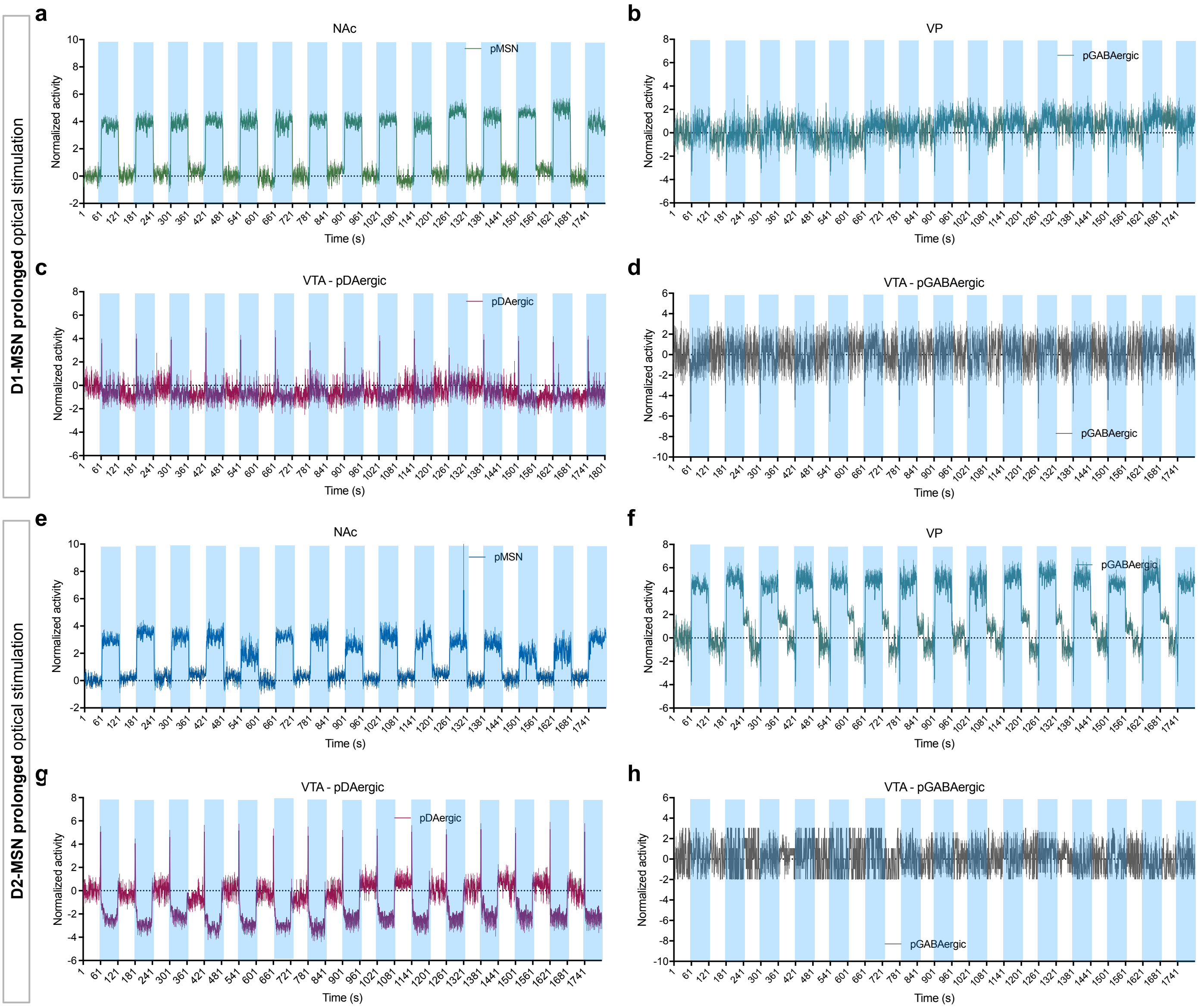

Supplementary Figure 8. Electrophysiological responses of NAc, VP and VTA during 30 minute protocol of prolonged stimulation. A 30min session with 60s stimulation (12.5ms pulses at 40 Hz) every 2min (which is equivalent to CPP test) was recorded, in order to evaluate the neuronal responses to repetitive stimulations. (**a**) Temporal variation of NAc pMSNs activity with D1-MSN prolonged stimulation; note the increase in activity during optical stimulation (n=9 cells/5mice). (**b**) Temporal variation of VP neuronal activity with D1-MSN brief stimulation; note the decrease in activity in the first 1-3s of optical stimulation and normalization thereafter (n=9 cells/5mice). (**c**) Temporal variation of VTA pDAergic neuronal activity with D1-MSN prolonged stimulation; note the increase in activity in the first second of optical stimulation and normalization thereafter (n=10 cells/6mice). (**d**) Temporal variation of VTA pGABAergic neuronal activity with D1-MSN prolonged stimulation; note the decrease in activity in the first 1-3s of optical stimulation and normalization thereafter (n=3 cells/6mice). (**e**) Temporal variation of NAc pMSNs activity with D2-MSN prolonged stimulation showing the increase in activity during optical stimulation (n=10 cells/5mice). (**f**) Temporal variation of VP pGABAergic neuronal activity with D2-MSN prolonged stimulation; note the decrease in activity during the first 2s of optical stimulation, and significant increase in the remaining stimulation period (n=10 cells/5mice). (**g**) Temporal variation of VTA pDAergic neuronal activity with D2-MSN prolonged stimulation. pDAergic neurons increase activity during the first 2s of optical stimulation, and then present a substantial decrease in activity in the remaining stimulation period (n=9 cells/5mice). (**h**) Temporal variation of VTA pGABAergic neuronal activity with D2-MSN prolonged stimulation. No significant effect of optical stimulation was found (n=3 cells/5mice).
Statistical significance is provided in Table 1 and Table 2 in Supp. Material.

Supplementary Figure 9

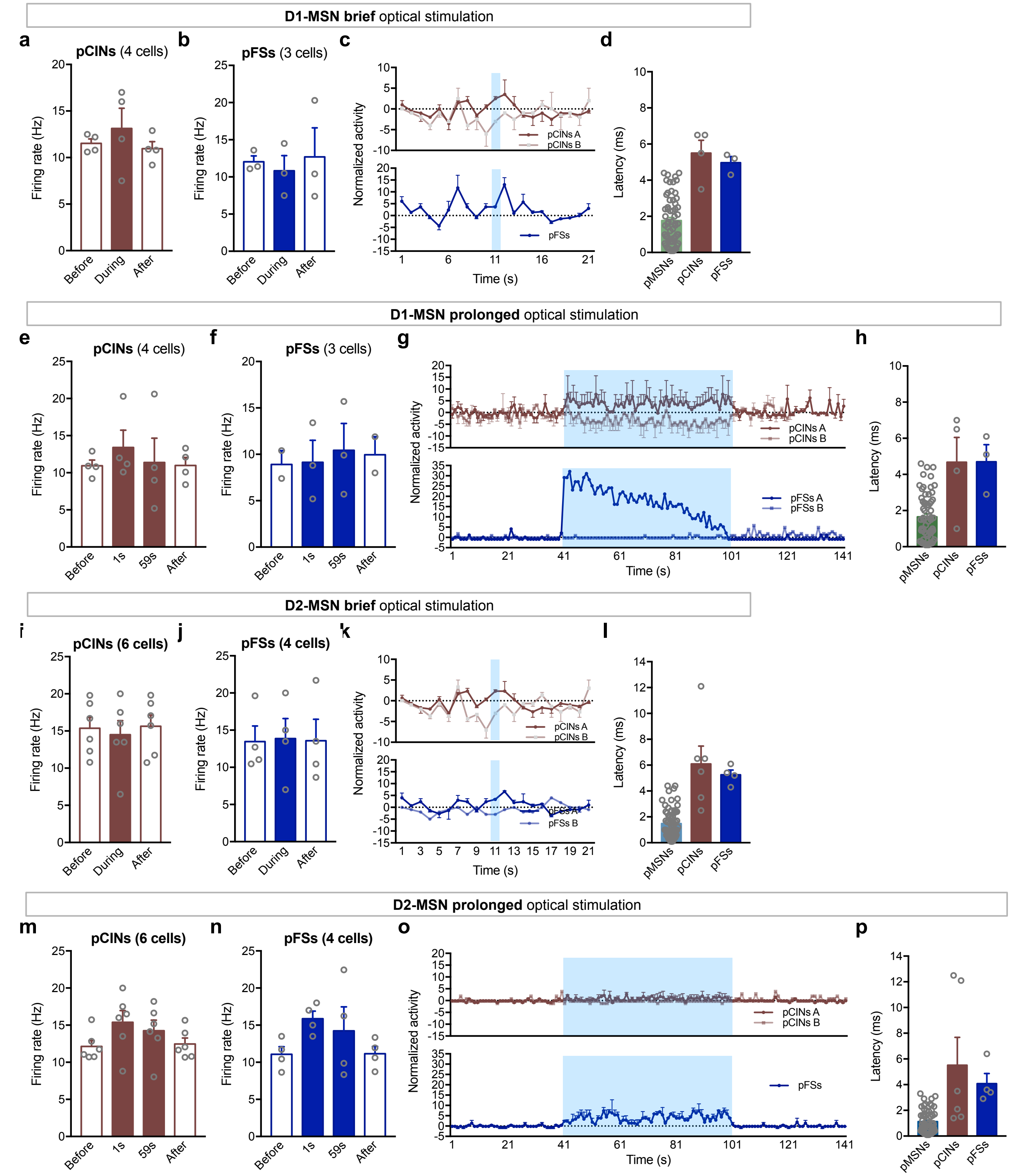

Supplementary Figure 9. Electrophysiological response of NAc neurons to brief and prolonged stimulation of D1- or D2-MSNs. Firing rate of pCINs (**a**) and pFS (**b**) before, during and after brief D1-MSNs optogenetic stimulation (1s, 40Hz, 12.5 ms pulses). (**c**) Temporal variation of pCINs and pFS activity; blue stripe represents optogenetic stimulation. (**d**) Latency to fire of different accumbal cells in response to brief D1-MSNs optogenetic stimulation. Firing rate of pCINs (**e**) and pFS (**f**) before, during and after prolonged D1-MSNs optogenetic stimulation (60s, 40Hz, 12.5 ms pulses). (**g**) Temporal variation of pCINs and pFS activity; blue stripe represents optogenetic stimulation. (**h**) Latency to fire in response to prolonged D1-MSNs optogenetic stimulation. Firing rate of pCINs (**i**) and pFS (**j**) before, during and after brief D2-MSNs optogenetic stimulation (1s, 40Hz, 12.5 ms pulses). (**k**) Temporal variation of pCINs and pFS activity; blue stripe represents optogenetic stimulation. (**l**) Latency to fire in response to brief D2-MSNs optogenetic stimulation. Firing rate of pCINs (**m**) and pFS (**n**) before, during and after prolonged D2-MSNs optogenetic stimulation (60s, 40Hz, 12.5 ms pulses). (**o**) Temporal variation of pCINs and pFS activity; blue stripe represents optogenetic stimulation. (**p**) Latency to fire in response to prolonged D2-MSNs optogenetic stimulation. $n_{D1\text{-}ChR2}$=4 mice (n_{pCIN}=4 cells, n_{pFS}= 3 cells); $n_{D2\text{-}ChR2}$=4 mice (n_{pCIN}=6 cells, n_{pFS}= 4 cells). Data are represented as mean ± SEM.

Supplementary Figure 10

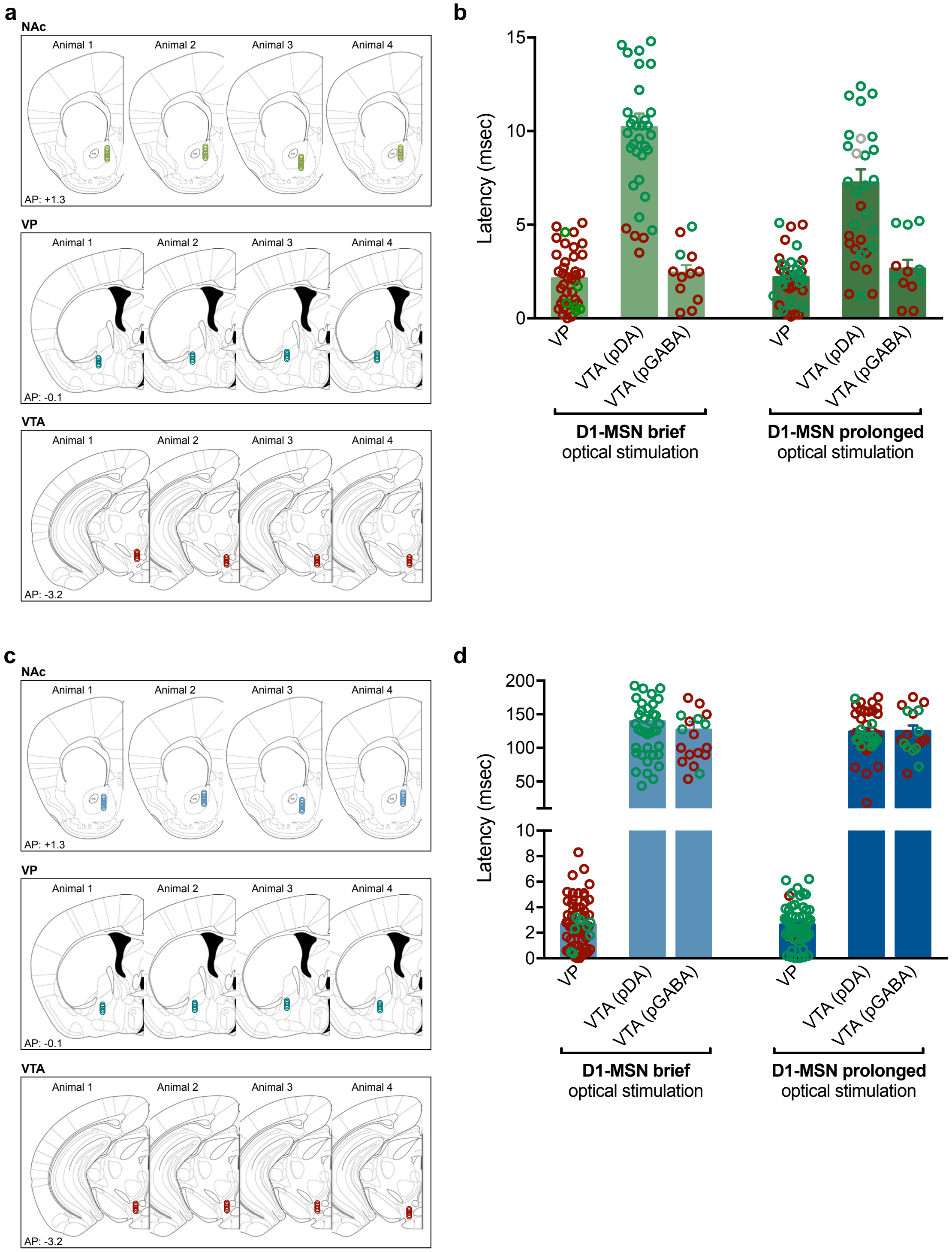

Supplementary Figure 10. Electrophysiological correlates of ventral pallidum and ventral tegmental area. (**a**) Placement of recording electrodes in the NAc, VP and VTA of D1-cre mice. (**b**) Latency to fire of VP and VTA neurons in response to brief (1s) or prolonged (60s) NAc D1-MSNs stimulation. (**c**) Placement of recording electrodes in the NAc, VP and VTA of D2-cre mice. (**d**) Latency to fire of VP and VTA neurons in response to brief (1s) or prolonged (60s) NAc D2-MSNs stimulation. In b and c, depicted in green are the neurons that increase activity in response to D1-MSN stimulation; in red are those that are inhibited (20% difference from baseline activity, as described in the methods). $n_{D1\text{-}ChR2}$=4 mice ($n_{pGABA\ VP}$=40 cells, $n_{pDA\ VTA}$= 35 cells, $n_{pGABA\ VTA}$=12 cells); $n_{D2\text{-}ChR2}$=4 mice ($n_{pGABA\ VP}$=62 cells, $n_{pDA\ VTA}$= 42 cells, $n_{pGABA\ VTA}$=20 cells). Data are represented as mean ± SEM.

Supplementary Figure 11

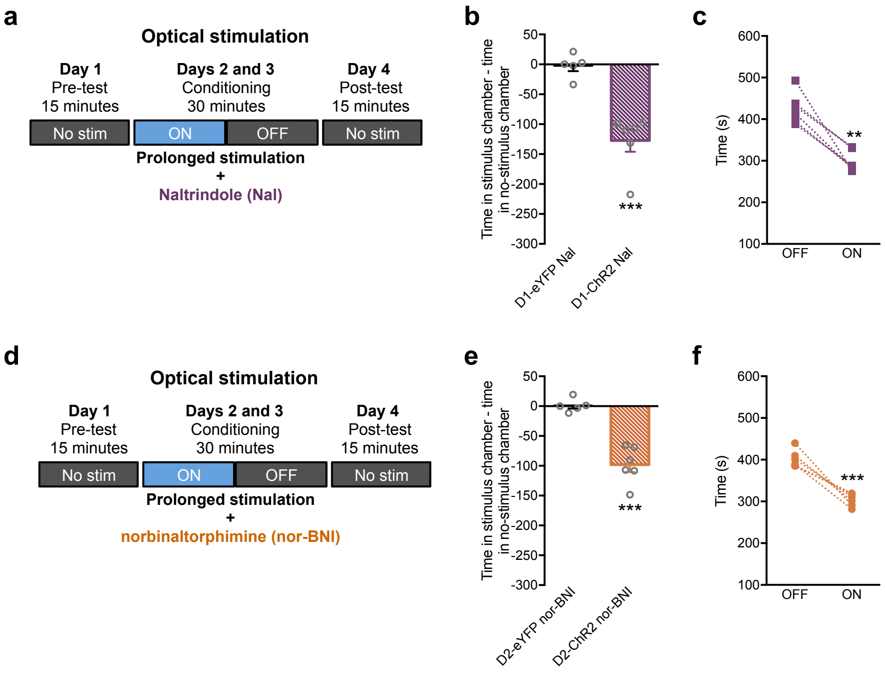

Supplementary Figure 11. Control experiments for optogenetic modulation with opioid receptors antagonists delivery. (**a-c**) Injection of DOR antagonist (naltrindole, nal, 0.1µg/0.5µl) in the VTA of D1-cre mice, 20 minutes prior to CPP conditioning, had no effect on the D1-MSN prolonged stimulation-induced aversive behaviour ($n_{D1\text{-}eYFPveh}$=5, $n_{D1\text{-}eYFPnal}$=5, $n_{D1\text{-}ChR2veh}$=6, $n_{D1\text{-}ChR2nal}$=6). (**d-f**) Injection of KOR antagonist (norbinaltorphimine, Nor-BNI, 1µg/0.5µl) in the VP of D2-cre mice, 20 minutes prior to CPP conditioning, had no effect on the D2-MSN prolonged stimulation-induced aversive behaviour ($n_{D2\text{-}eYFPveh}$=5, $n_{D2\text{-}eYFPnor\text{-}BNI}$=5, $n_{D2\text{-}ChR2veh}$=6, $n_{D2\text{-}ChR2veh}$=6). **p<0.01, ***p<0.001. Data is represented as mean ± SEM.
